# Analysis of the protective efficacy of approved COVID-19 vaccines against Omicron variants and the prospects for universal vaccines

**DOI:** 10.3389/fimmu.2023.1294288

**Published:** 2023-11-27

**Authors:** Keda Chen, Ling Zhang, Zhongbiao Fang, Jiaxuan Li, Chaonan Li, Wancheng Song, Zhiwei Huang, Ruyi Chen, Yanjun Zhang, Jianhua Li

**Affiliations:** ^1^ Shulan International Medical College, Zhejiang Shuren University, Hangzhou, China; ^2^ School of Medical Technology and Information Engineering, Zhejiang Chinese Medical University, Hangzhou, China; ^3^ Department of Virus Inspection, Zhejiang Provincial Center for Disease Control and Prevention, Hangzhou, China

**Keywords:** SARS-CoV-2, COVID-19, Omicron variants, vaccine effectiveness, convergent evolution, universal vaccines

## Abstract

By the end of 2022, different variants of Omicron had rapidly spread worldwide, causing a significant impact on the Coronavirus disease 2019 (COVID-19) pandemic situation. Compared with previous variants of severe acute respiratory syndrome coronavirus 2 (SARS–CoV-2), these new variants of Omicron exhibited a noticeable degree of mutation. The currently developed platforms to design COVID-19 vaccines include inactivated vaccines, mRNA vaccines, DNA vaccines, recombinant protein vaccines, virus-like particle vaccines, and viral vector vaccines. Many of these platforms have obtained approval from the US Food and Drug Administration (FDA) or the WHO. However, the Omicron variants have spread in countries where vaccination has taken place; therefore, the number of cases has rapidly increased, causing concerns about the effectiveness of these vaccines. This article first discusses the epidemiological trends of the Omicron variant and reviews the latest research progress on available vaccines. Additionally, we discuss progress in the development progress and practical significance of universal vaccines. Next, we analyze the neutralizing antibody effectiveness of approved vaccines against different variants of Omicron, heterologous vaccination, and the effectiveness of multivalent vaccines in preclinical trials. We hope that this review will provide a theoretical basis for the design, development, production, and vaccination strategies of novel coronavirus vaccines, thus helping to end the SARS-CoV-2 pandemic.

## Introduction

In 2019, a novel coronavirus pneumonia caused by severe acute respiratory syndrome coronavirus 2 (SARS-CoV-2) became prevalent worldwide (1) and was declared a pandemic on March 11, 2020 (2). According to World Health Organization (WHO) statistics, as of October 12, 2023, there were approximately 771 million global infections of SARS-CoV-2, resulting in a death toll of approximately 6.961 million (3). Approximately 1.349 billion vaccines have been administered, imposing significant health and economic burdens worldwide. As the epidemic progressed, concerning variants emerged, including BA.5, BF.7, XBB.1.5, and XBB.1.16. By August 2023, EG.5.1 accounted for 18% of global infections, while XBB accounted for 78% of infections (XBB.2.3: 18%, XBB.1.9: 15%, XBB.1.16: 16%, XBB.1.5: 31%, XBB: 2%, XBB.2.75: 1%) (4). Mixed infections or reinfections were also prevalent, including BA.4/5, BF.7, BQ, BQ.1.1, CH.1.1, and others. These new mutants exhibit more mutations in the spike protein receptor binding domain (RBD) than BA.2, resulting in increased spread and mortality of COVID-19 (5, 6). They also possess enhanced immune escape abilities compared with the original strain (7–9). As the global novel coronavirus epidemic persists, vaccines serve as an economical and effective means to prevent further infections, fundamentally curbing the further spread or resurgence of the epidemic. However, the new mutants have demonstrated a robust immune response and immune escape capabilities against vaccines developed based on the original strain sequence (such as CoronaVac and ZF2001). In addition, studies have shown evidence of dynamic diversification of the novel coronavirus population found in most patients, indicating that immune-deficient systems can select different immune escape mutants in specific tissues and organs of patients. Compared with other viruses, the novel mutants utilize an immunodeficiency system to induce a more intricate pattern of mutation (7).

Vaccine effectiveness is a crucial indicator to determine whether a vaccine can be approved for marketing, and it assesses the level of protection provided to the entire community (8, 9). Evaluating the effectiveness of vaccines involves three main aspects: The strength of the immune response in both humoral and cellular immunity, the duration of the vaccine-induced immune response, and the response level of the elderly population to the vaccine (10–12). To establish the effectiveness of vaccines, researchers typically conduct phase III clinical trials to examine their impact on reducing the incidence and mortality of COVID-19 (13, 14). However, the emergence of different Omicron variants has posed new challenges to global efforts to control the pandemic. Meanwhile, new vaccines have emerged, including aerosol Ad5-nCoV, SCTV01E, and BNT162b4 vaccines, among others. The number and types of vaccinations are also different. Therefore, although we have previously conducted a series of summaries on the effectiveness of vaccines already on the market for different SARS-CoV-2 mutants such as variants of concern (VOCs: Beta, Alpha, Gamma, Delta, Omicron BA.1) (15), it is still necessary to summarize the effectiveness of new vaccines.

This review summarizes the efficacy of recently approved vaccines against various variants of the Omicron strain. We introduce the convergent evolution and pedigree development of Omicron mutants and provide information on the lineages and key mutation sites of different variants of Omicron. Then, we summarize the latest progress of different types of vaccines. On this basis, the effectiveness of different subvariants of Omicron was evaluated for the latest vaccination program. In addition, because of the continuous mutation of the virus, there is an urgent need to develop a universal vaccine for these mutant strains to reduce the adverse effects caused by mutations. Therefore, we also discuss the progress of several universal vaccines worthy of further study. Our goal is to provide a scientific reference and source of inspiration for the development, production, and vaccination strategies for new SARS-CoV-2 mutants, thus helping to end the COVID-19 pandemic.

## Convergent evolution of Omicron subvariants

Marc Johnson believes that the Omicron mutant strain is in the era of great convergence (5). Although the sub-variants of Omicron have diverged during the mutation process, their sub–variants have gained mutations in the spike protein, such as at positions 346, 444, 452, 460, and 486 (5, 16). The mutations in the RBD converge at the same site. The binding affinity between SARS-CoV-2 and Angiotensin Converting Enzyme 2 (ACE2) plays a crucial role in the transmission of the virus (17, 18). The binding ability of human ACE2 is assessed by the inhibition efficiency of hACE2 on the pseudovirus, and if the inhibition efficiency is high, it indicates that the binding ability of hACE2 to the virus is strong (19).

The mutant sub-strains continue to evolve in different directions, and the complexity of their spike proteins are constantly being updated. The BA.5 sub-strain differs from the BA.2 sub–strain in the RBD, specifically the mutations L452R, F486V, and R493Q (20). Building upon BA.5, the BF.7 sub-strain has additional mutations related to the convergent evolution of K444T and R346T. Similarly, the BQ.1 sub-strain, which used to be dominant, has spike proteins with K444T and N460K mutations compared to BA.5. BQ.1.1 is a new mutant of BQ.1. Compared with BQ.1, it has spike protein mutation R346T. XBB is a recombinant strain of BJ.1 and BM.1.1.1, in which BJ.1 is produced by the BA.2.10 mutation and BM.1.1.1 by a series of mutations of BA.2.75. XB ‘s N-terminal structure has five mutations, and its RBD has nine mutations, including R346T, N460K, and F486S. The XBB sub–strain XBB.1 has an additional spike protein mutation, G252V, ORF8 has G8stop compared with XBB, and XBB1.5 shares the L368 I, V445 P, N460K and F490 S mutations. The difference is that XBB has the F486S mutation, and XBB.1.5 has the F486P mutation (21). Compared with XBB and XBB.1, XBB.1.5 shows a higher affinity with ACE2 (21). Subsequently, XBB.1.16 was discovered to have two alternative mutations in the spike protein compared with XBB.1.5: E180V and T478R (22).

Neutralizing antibodies frequently target the spike proteins with L452R, F486V, and R493Q mutations, although spike proteins with L452R and F486V mutations can evade most cross-reactive neutralizing antibodies (16, 23). Specifically, L452 is primarily resistant to two and three types of RBD monoclonal antibodies (mAbs), and L452R is relatively more harmful. L452R is found in BA.5, XBB sub-mutants, and other variants. Zhang et al. discovered that the L452R mutation increased the dispersibility, infectivity, and enhanced S protein cleavage of SARA-CoV-2 (24). By contrast, the F486V mutation negatively affects the neutralization activity of several class 1 and class 2 RBD mAbs. Compared with F486V, the F486S mutation adversely impacts the binding of class I and III mAbs and ACE2 (20). Finally, antibody avoidance is not primarily dependent on the R493Q mutation. However, it can enhance affinity for hACVE2 by reversing or restoring the affinity to receptors, thus providing significant advantages for ACE2 binding (20). The slightly reduced affinity between XBB and XBB.1 and ACE2 might be attributed to the presence of mutations F486S and R493Q (24, 25).

K444T, a mutation that exists in BF.7, BQ.1, and BQ.1.1, etc., enables the viruses to escape class III antibody recognition (26), possibly because the mutation of lysine to threonine makes the side chain shorter and uncharged, which in turn damages the interaction between this residue and the mAbs targeting this site, such as SP1-77 and LY-CoV1404. Similar to K444T, the V445P substitution in XBB and its variants also has the same effect. V445P substitution can cause steric hindrance and destroy the hydrogen bond with mAbs, resulting in the loss of antibody neutralization (25). Finally, the presence of R346T was observed in the mutant XBB and its sub-mutants, BF.7 and BQ.1.1, which are capable of evading E2.1- and E2.2-neutralizing antibodies (nAbs). Compared with BQ.1, BQ.1.1 possesses an additional R346T mutation, resulting in an increased ability to evade antibodies against three types of RBD mAbs. Moreover, both BQ.1 and BQ.1.1 sub-varieties exhibit enhanced resistance to neutralization, primarily attributed to the N460K mutation (26). Adding R346T and K444N based on BA.5 can result in the escape from most nAbs and a strong binding ability to ACE2. This study demonstrates the potential for the mutant strain to mutate further and generate a self-contained variant with both a robust ACE2-binding capability and a high escape potential (16). XBB and XBB.1 are commonly encountered strains that evade the humoral immune response. In their study, Cao et al. measured the Geometric Mean Titer (GMT) of XBB and XBB.1 infection with BA.5 after three doses of CoronaVac inoculation. The NT50 values for XBB and XBB.1 were 27 and 26, respectively, compared to SARS-CoV-1 from 2003, which had an NT50 of 29. Similar findings were observed in their other group as well. This indicates that the antigenic distance of XBB is greater than SARS-CoV-1, indicating a significant antigenic drift and potential serum conversion (16). Yamasoba discovered that the dissociation constant of XBB.1.16RBD with the human ACE2 receptor was 2.4 times higher than that of XBB.1.5RBD, suggesting that XBB.1.16RBD with ACE2 was lower than that of XBB.1.5 (22). However, in the pseudovirus neutralization experiment, both strains exhibited similar infectivity. This led the researchers to speculate that the discrepancy might be due to the peak conformation of the spike protein monomer and trimer (22).

## Vaccine development

**Figure 1:**
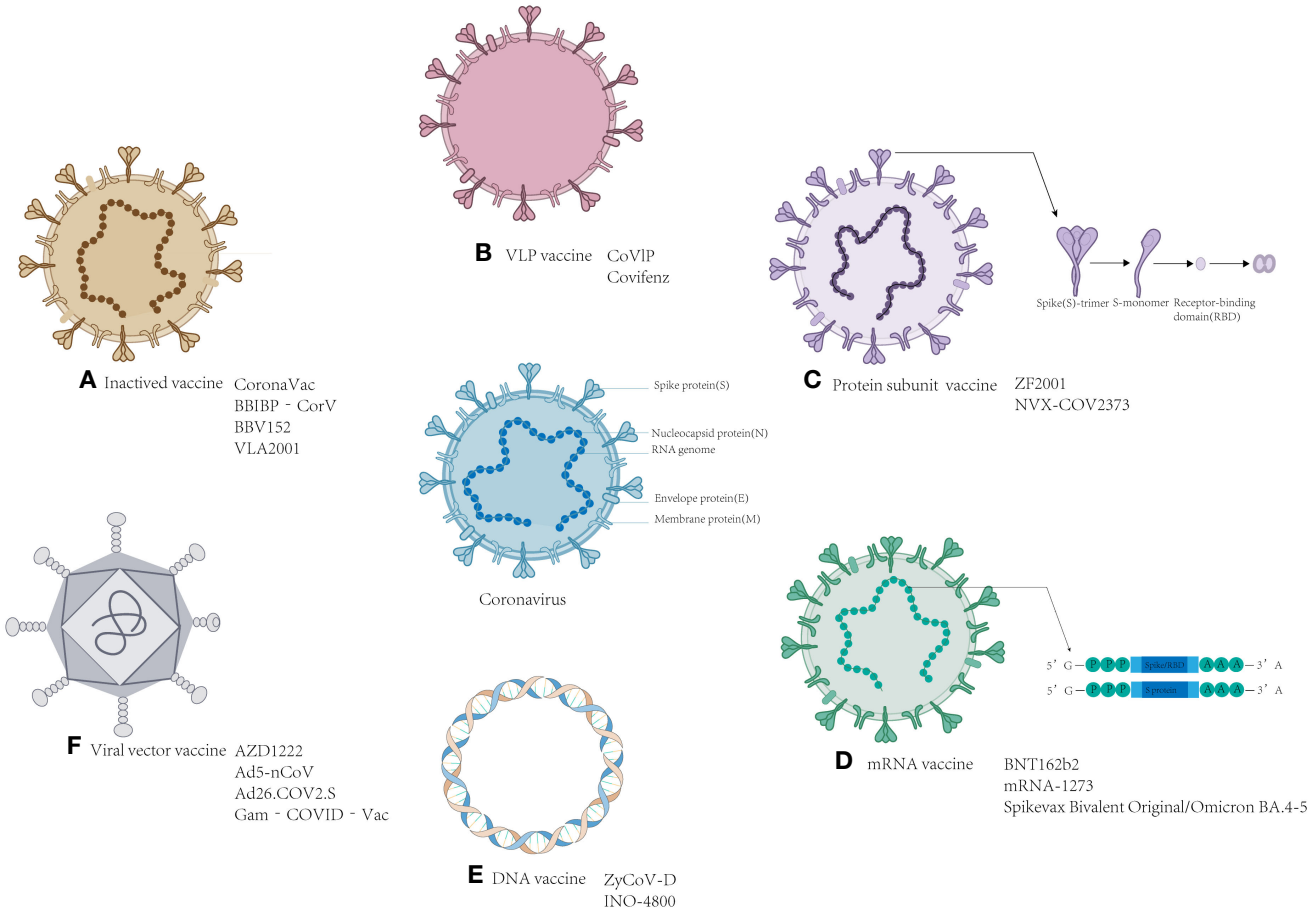
Schematic representation of the various SARS-CoV-2 vaccines. Located in the middle of the picture is the structure of coronavirus. The structure and the name of the vaccines: **(A)** Inactivated vaccine; **(B)** VLP vaccine; **(C)** Protein subunit vaccine; **(D)** mRNA vaccine; **(E)** DNA vaccine; and **(F)** viral vector vaccine.

Vaccination plays a crucial role in stimulating the immune system to mount an antigen response (27), and as of March 2023, the WHO has approved 50 vaccines for marketing. These vaccines encompass a variety of types, including mRNA vaccines, DNA vaccines, inactivated vaccines, virus particle-like (VPL) vaccines, viral vector vaccines, and protein subunit vaccines, all of which are specifically designed to combat COVID-19. Multivalent and heterologous vaccinations have become increasingly prevalent as the epidemic progresses in different regions. Our previous studies have extensively covered these vaccines, providing definitions, development methods, and a comprehensive review of their advantages and disadvantages (15). In this review, we conducted supplementary research on different types of vaccines currently on the market. We gathered current data on the effectiveness of the different types of these vaccines. Additionally, we summarized the advances made in several universal vaccines that show promise for further investigation, contributing theoretical value to the development of universal vaccines. The research and development institutions and the progress in the development of various types of vaccines are shown in **Table S1** and **Figure 1**.

## mRNA vaccines

mRNA vaccines are a promising therapeutic strategy with high vaccine efficacy. They have several advantages, including cost-effectiveness and relatively low severity of side effects. Unlike the production of traditional vaccines (killed or attenuated pathogens) (28), in which cells or viruses need to be cultured, mRNA vaccines are mainly synthesized *in vitro*, which can achieve large-scale and short–cycle production, making them common in epidemiology and other fields (29).

As of March 2023, nine mRNA vaccines have received marketing approval. mRNA technology is a novel approach that delivers nucleic acid molecules encoding antigens to target cells via a carrier. This stimulates the production of the antigen proteins, triggering immune responses and rapidly inducing both humoral and cellular immunity (30). The most widely used mRNA vaccines are BNT162b2 and mRNA-1273.

In a large-scale vaccination study involving 10,125 participants (two doses of BNT162b2 were administered 6 months ago), 5,081 participants received a third dose of the BNT162b2 vaccine, while 5,044 participants received a placebo. During the 2.5-month median follow-up period, the vaccine efficacy of the third dose of BNT162b2 against the novel coronavirus was found to be 95.3% compared with that of the second dose (31). Based on monovalent mRNA, more novel bivalent mRNA vaccines have emerged, such as the mRNA-1273.214 vaccine encoding Wuhan-1 and BA.1 antigens and the mRNA-1273.22 vaccine encoding BA.4/5 spike protein. The bivalent mRNA vaccines produced a higher GMT than the monovalent mRNA (32). In addition, Davis-Gardner’s group showed that the neutralizing antibody titers (GMT, NT50, BA.5: 250, BQ.1.1: 73, XBB: 37) produced by one dose of a bivalent mRNA vaccine (containing mRNA encoding an ancestral spike and mRNA encoding the omicron BA.5 spike protein) were higher than those produced by two doses of the monovalent mRNA vaccine (GMT NT50, BA. 112, XBB: 96) (33). These serological data demonstrate the benefits of bivalent vaccines in the population.

As of August 31, 2022, the bivalent vaccines BNT162b2 produced by Pfizer-BioNTech and mRNA-1273 produced by Moderna were officially approved by the US FDA. From August 31 to October 23, approximately 14.4 million individuals aged 12 and above received the bivalent mRNA vaccine booster produced by BioNTech, and about 8.2 million people over 18 years old were vaccinated with the bivalent mRNA booster produced by Moderna. During this period, there were only 5542 reports in the Vaccine Adverse Event Reporting System (VAERS), with non-serious reports accounting for 95.5% (34). However, only 914 vaccine adverse events were reported in the VAERS after inoculation with BNT162b2 produced by Pfizer, with non-serious reports accounting for 91.6% (34). In terms of the proportion of non-serious reports, the administration of bivalent mRNA vaccines had a very small impact on the population’s health, and serious adverse events were rare.

## Inactivated vaccines and attenuated live vaccines

Inactivated vaccines typically include the entire pathogen, providing a range of proteins for immunization. These vaccines exhibit stable expression (27) of dependent antigen epitopes, facilitating immune recognition. The novel coronavirus contains multiple structural proteins, including spike (S), membrane (M), envelope (E), Nucleocapsid (N), and other auxiliary proteins such as ORFs (3a, 6, 7a, and 7b) (35), resulting in antibodies against at least eight proteins in the serum of patients. However, inactivated vaccines often require multiple doses or the addition of adjuvants to elicit a humoral immune response. Additionally, their yield may be subject to the production capacity of the virus in cell culture and the requirements of biosafety level 3 production facilities (36, 37). Attenuated live vaccines use the weakened virus as the antigen and are generally grown in low-temperature non-human cells, and codons are optimized to achieve attenuated effects (38–41). However, in some cases, live attenuated vaccines might have restored activity (38, 42, 43).

In terms of the novel coronavirus, as of March 2023, a total of 11 inactivated vaccines have received approval. CoronaVac, BBIBP-Corv, WIBP-Corv, and others have been widely utilized. CoronaVac has been granted marketing approval and is administered in a two-dose regimen with a 14-day interval. Before April 7, 2021, CoronaVac had been administered in over 2 billion doses across 169 countries, establishing it as the most extensively employed SARS-CoV-2 vaccine worldwide. Consequently, CoronaVac played a crucial role during the epidemic (39, 42). McMenamin et al. evaluated the effectiveness of three doses of the CoronaVac vaccine in Hong Kong using an ecological study design first used in Israel. In mild or moderate diseases, the efficacy of the three-dose CoronaVac vaccine was 51.0% (95% CI, 39.6–60.4). Among the people who previously received two doses of CoronaVac, the third dose of CoronaVac increased the protection rate of those aged 20-59 years (35.7% [95% CI, 22.1-47.3]) and ≥ 60 years old (46.9% [95% CI, 29.6-60.6]). For severe or fatal cases, the protection rate of the vaccine in both the two-dose and three-dose CoronaVac regimens was 87.9 (79.5–93.3%) in individuals aged over 80 years. Additionally, the study noted that vaccination with a third dose of CoronaVac at all ages has additional benefits, and older people need to be vaccinated with three doses of CoronaVac to receive a high level of protection (44).

The inactivated vaccine primarily stimulates B lymphocytes to produce antibodies, while the T-cell response is comparatively weaker (45–47). Researchers, including Yanjun Zhang, found that the CoronaVac vaccine developed by Beijing Kexing, Zhongwei Biotechnology Co., Ltd. induced low levels of interferon gamma (IFN-γ) in participants (48). The Beijing Institute of Biological Products developed BBIBP-CorV. This inactivated vaccine can induce an excellent immune reaction (49). Meanwhile, T cells can induce responses against S, N, and E structural proteins of SARS-CoV-2 (50).

## Viral vector vaccines

Viral vector vaccines typically contain viral vectors, such as adenovirus (Ad) and the modified Ankara strain (MVA) (40). A viral vector vaccine has the advantage of inducing the innate humoral immune response and the cellular immune response (36). However, this strategy might reduce the effect because some individuals have preexisting immunity to the vector because of previous natural infections. Recombinant viral vectors and heterologous vaccination might overcome this challenge. Logunov et al. developed a heterologous COVID-19 vaccine consisting of a recombinant adenovirus type 26 (rAd26) vector and a recombinant adenovirus type 5 (rAd5) vector containing SARS-CoV-2 spike protein rAd26-S and rAd5-S genes. In tests, the vaccine elicited a robust cellular and humoral immune response (51).

As of March 2023, 9 of the 50 viral vaccines have been approved as non-replicating viral vector vaccines. The National Research Center for Epidemiology and Microbiology of Gamaleya developed the Sputnik Light and Sputnik V vaccines. Sukhikh et al. conducted a study using these vaccines among healthcare workers from November 26, 2020, to February 8, 2022. Throughout this timeframe, the vaccine demonstrated an effectiveness of 89.1% (86.9–91.0%), with 96.5% (75.0–99.5%) of the participants receiving three to four doses (52).

According to the WHO, the Ad5-nCoV vaccine has an efficacy of 58%, and the efficacy against severe novel coronavirus pneumonia was 92% in a phase 3 clinical trial of two doses of inactivated vaccine followed by one dose of aerosol Ad5-nCoV (53). Conversely, the control group received two doses of the inactivated vaccine followed by 0.5 ml of the same vaccine. The GMT of aerosol Ad5-nCoV to omicron BA.4/5 was 107.7 (95% C, 88.8–130.7), and the GMT of inactivated vaccine group to Omicron BA.4/5 was 17.2 (95% C, 16.3–18.2) (54). It can be seen that the GMT of aerosol Ad5-nCoV was 6.29 times higher than that of the inactivated group, and the effect of aerosol Ad5-nCoV was better. Jin et al. conducted a serum test on individuals who received two doses of CoronaVac followed by one dose of atomized Ad5 (low-dose group: 0.1 ml, high-dose group: 0.2 ml). The geometric GMT method was used to detect the nAbs in the two groups. On the 28th day, the GMT of the nAbs in the two groups against Omicron BA.4/5 pseudovirus was 149.58 (95% CI 101.03–221.45) and 158.52 (95% CI 111.36–225.66), respectively. The serum positive rate of nAbs against Omicron BA.4/5 at low or high doses was 97.4% (55). Tang et al. studied the injection of three doses of CoronaVac followed by one dose of aerosol Ad5–nCoV or one dose of intramuscular Ad5-nCoV. After 28 days, the GMT of the wild-type strain induced by one dose of aerosol Ad was (CI 539.7–837.7), while the GMT induced by one dose of intramuscular Ad5-nCoV booster was 582.6 CI (505.0–672.2). Compared with the Ad5-nCoV serum-neutralizing antibody, the GMT induced by the aerosol version was significantly increased. The findings suggest that oral aerosol Ad5-nCoV can elicit a robust adaptive immune response (56).

DelNS1-RBD4N-DAF is an intranasal candidate vaccine based on a live attenuated influenza virus (by Chen Honglin from the University of Hong Kong). This vaccine is derived from a previously studied attenuated influenza virus (live attenuated influenza virus, LAIV). By deleting the NS1 gene of the influenza virus (DelNS1) and introducing adaptive mutations, an LAIV can be developed into an influenza vaccine due to its ability to replicate in chicken embryos and MDCK cells. The vaccine inserts the SARS-CoV-2 RBD in the NS1 deletion site of the influenza virus. Additionally, it introduces four glycosylation sites and cell membrane anchoring proteins in the RBD sequence. In a hamster experiment, it was found that compared with the BioNTech BNT162b2 mRNA vaccine, this vaccine induced a high level of neutralizing antibody titers against Omicron BA.1 and BA.2 and stimulated the hamsters to produce strong CD4 + and CD8 + T cells. It is worth mentioning that the vaccine can be used to create a bifunctional vaccine against influenza and novel coronaviruses to prevent influenza and SARS-CoV-2 virus infection (57).

## Protein subunit vaccines

A protein subunit vaccine comprises protein fragments that can elicit additional cellular or humoral immune regulation, trigger Th cell responses and germinal center responses, and enhance the protection rate (58). Compared with the inactivated vaccine, the protein subunit vaccine only includes the essential antigenic portion of the pathogen to augment immune protection, making it safer and more reliable (40). Protein subunit vaccines are usually divided into recombinant S protein vaccines and RBD vaccines. The expression of the spike protein is challenging and less efficient than the RBD vaccine. Although the RBD protein has a smaller molecular weight, it lacks other neutralizing epitopes present in the full-length spike protein, making it susceptible to antigen drift (59).

Bhiman et al. conducted a study to measure serum antibodies in individuals who received three doses of the protein vaccine NVX CoV2373 (Novavax). One month after receiving the three doses of the NVX CoV2373 vaccine, the GMTs of Omicron BA.1 and BA.4/BA.5 in plasma were 1197 and 582, and the GMTs of BA.4/BA.5 in plasma were 1078 and 667, respectively. The reactions triggered by three doses of NVX CoV2373 and three doses of BNT162b2 to Omicron BA.4/5 were similar. During the Omicron BA.4/5 epidemic, NVX CoV2373 has potential value as a reinforcing agent (60, 61).

## DNA vaccines

DNA vaccines are typically produced using plasmid DNA that encodes pathogen proteins. Gurunathan et al. demonstrated that DNA vaccines can induce both humoral and cellular immunity (62). Among the 50 vaccines approved for the novel coronavirus as of March 2023, one DNA vaccine called ZyCoV-D has been authorized for use. The ZyCoV-D vaccine contains plasmid DNA that encodes the SARS-CoV-2 spike protein S gene and the signal peptide gene (63). This vaccine is currently undergoing phase 3 clinical trials in 49 hospitals across India. The trials are being conducted using a multi-center, double-blind, randomized, controlled design, including 28216 participants (64). The vaccine demonstrated an efficacy of 66.6% (95% CI 47.6–80) against COVID-19 (65). Furthermore, our previous studies have highlighted the storage advantages of DNA vaccines (15). Proper vaccine storage is crucial for maintaining the quality of vaccines, and DNA vaccines have better stability compared with other types of vaccine. The ZyCoV-D vaccine can be stored at temperatures between 2 and 8°C and can be stable at 25°C for at least 3 months; thus, it is more suitable for use in epidemic areas (65).

## Virus-like particle vaccines

A virus-like particle (VLP) is a multi-protein structure that lacks the virus’s genetic material, thus enhancing the safety of the vaccine produced by these particles. The protein in the VLP vaccine is a self-assembled viral structural protein, which mimics the conformation of the natural SARS-CoV-2 virus. Furthermore, as mentioned in our previous article, the presence of the S protein on the surface of VLPs enables them to bind to ACE2 receptors just like the virus and enter the host cells. It directly cross-links with and activates B cells, resulting in immunogenicity and the induction of high neutralizing antibody titers (15, 27, 66).

As of March 2023, Covifenz (Medicago), a VLP vaccine, has been approved among the 50 vaccines worldwide. On February 24, 2022, Health Canada approved Covifenz, making it the first plant-based vaccine approved for the treatment of SARS-CoV-2. The vaccine is administered in a two-dose regimen with a 21-day interval (phase III clinical trial lot number: NCT05040789). Each dose of the Covifenz vaccine contains 3.75 μg of SARS-CoV-2 VLP and 0.25 ml of AS03 adjuvant (67). Covifenz provides substantial protection against COVID–19, with 71% effectiveness in the population aged 18-64 (68).

## Universal vaccines

In recent years, the development of mRNA vaccines has emerged as a successful approach to combat epidemics, and such vaccines have played a significant role in population immunization. However, as new subtypes continue to emerge, the effectiveness of monovalent and bivalent mRNA vaccines has been investigated. Barouch et al. conducted a study comparing the neutralizing antibody titer and CD4+ or CD8+ T-cell response to BA.5 between two vaccines. Their findings revealed that although there was no significant difference in the immune responses (69, 70), the bivalent mRNA vaccine demonstrated improved protection against symptomatic diseases in individuals aged 18 to 49 years in the short term (71, 72). Increasing the number of vaccines or their dosage provides the most benefit to people who need to prevent serious diseases, are older, have low immunity, and have multiple diseases and high risks (71). Therefore, a key question is whether current vaccines can effectively deal with future mutations of SARS-CoV-2.

**Figure 2:**
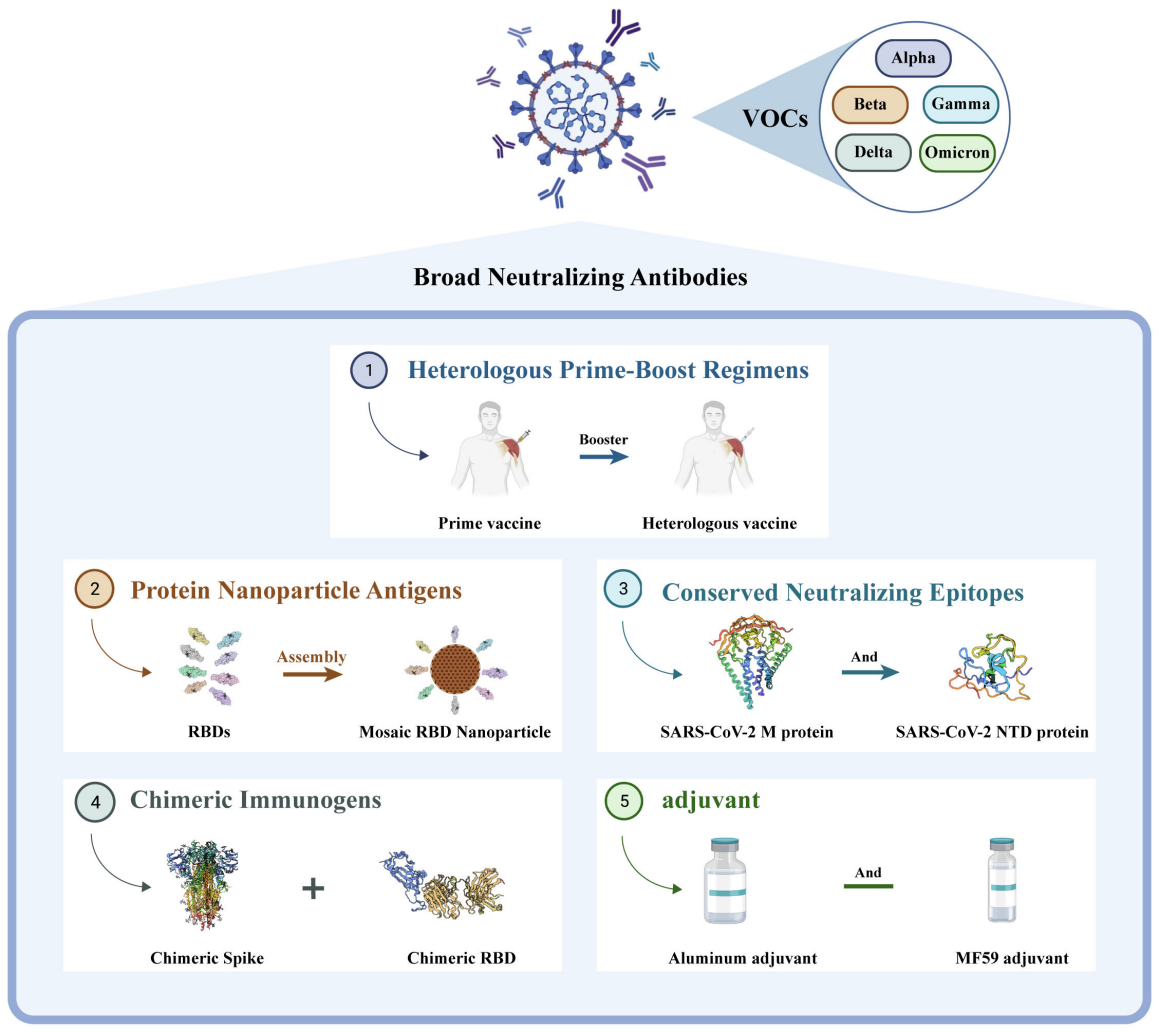
Different directions in the development of universal vaccines. They are ① heterologous prime-boost vaccination regimens, ② construction of protein nanoparticle antigens, ③ utilization of conserved neutralizing epitopes, ④ design of chimeric immunogens, and ⑤ new adjuvant.

Based on the current emergence of new mutant strains exhibiting strong immune evasion capabilities (73), the development of universal vaccines could address the challenges posed by future mutant strains and achieve a broader range of neutralization effects. Currently, the design concepts for universal vaccines primarily revolve around heterologous inoculation schemes, construction of chimeric immunogens, design of protein nanoparticle antigens, utilization of conserved neutralizing epitopes, and novel adjuvants (see **Figure 2**). This review discusses several noteworthy studies on universal vaccine design with the hope that these findings can inspire the further development of universal vaccines.

Recently, the aerosol Ad5-nCoV and S trimer protein vaccine (SCTV01E) were included as emergency vaccines. Inhaling 0.1 or 0.2 ml of aerosol Ad5-nCoV as a heterologous booster can significantly enhance the serum nAb response. Compared with intramuscular injection, inhalation vaccination can protect against infection in the airway mucosa and activate the resident memory B and T cells in the respiratory mucosa (74). In the early stage, memory B and T cells can hinder virus replication and transmission more effectively (75). The nebulized Ad5-nCoV vaccine also elicits stronger cellular immunity, which aids in eliminating cells infected with SARS-CoV-2 (76). It is safe and immunogenic as a heterologous vaccine in vaccinated individuals, inducing robust humoral and antibody responses. The SCTV01E S trimer protein vaccine consists of four variants: Alpha, Beta, Delta, and Omicron BA.1 (77). It employs a novel oil-in-water adjuvant of Th1 cells to offer enhanced protection against SARS-CoV-2. In individuals vaccinated with the mRNA vaccine, a clinical study demonstrated that 30 μg of the tetravalent protein vaccine SCTV01E was well tolerated and demonstrated a broader and more superior cross-neutralization ability against the new Omicron subvariant (78). Hence, aerosol Ad5-nCoV and SCTV01E might be promising tools for future responses to SARS-CoV-2 variants.

Scientists have developed a new mRNA vaccine called BNT162b4, which targets the conserved sequence of the non-spike protein. This vaccine can potentially enhance T-cell immunogenicity in different populations against SARS-CoV-2 infection as it can reduce the severity and duration of the disease (79). BNT162b4 encodes fragments of SARS-CoV-2 N, M, and ORF1ab proteins and is primarily used in combination with BNT162b2. BNT162b4 induces the response of multifunctional CD4 + and CD8 + T cells to various epitopes. In a hamster model experiment, BNT162b4 was found to reduce the severity of disease caused by different virus variants. Currently, BNT162b4 is being used in combination with the BA.4/BA.5 Omicron-updated bivalent BNT162b2. Another universal vaccine, the Span vaccine, induced more extensive immunity to various mutant strains in mice compared with the wild-type vaccine (Swt). Mice vaccinated with heterologous Span boosters were entirely protected against lethal Omicron variant infection (80). The Span vaccine achieves broad–spectrum activity by including conserved neutralization epitopes of the S protein that remained during the evolution of SARS-CoV-2. This allows it to neutralize various circulating strains and respond to future sub-variants.

## Effectiveness of approved vaccines against Omicron

**Figure 3:**
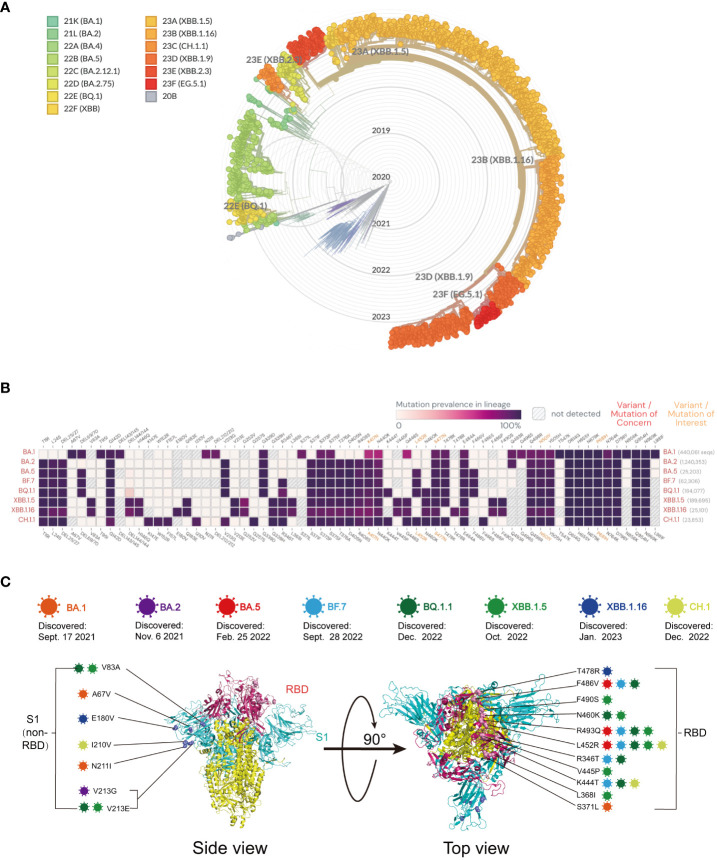
**(A)** Global phylogeny of severe acute respiratory syndrome coronavirus type 2 reveals the Omicron lineage. The Nextstrain platform provides a time-calibrated global phylogeny of severe acute respiratory syndrome coronavirus type 2 (https://nextstrain.org/ncov/gisaid/global). Different colors represent different Omicron variants observed and their variants of interest. The Omicron variant is detailed in the article. **(B)** Mutation prevalence across lineages. The Outbreak platform provides the S protein mutation prevalence across lineages. The figure describes in detail the mutation prevalence in the lineage of the VOCs. Orange indicates a mutation of interest, and red indicates a mutation of concern. Blank indicates that the mutation has not been detected. White to purple indicates the prevalence of the mutation in all sequences. **(C)** Schematic representation of the mutation sites on various mutant viruses. Schematic diagram of the SARS-CoV-2 spike protein structure and the mutation sites of the mutant strains in this review: BA.1 (orange), BA.2 purple), BA.5 (red), BF.7 (blue), BQ.1.1 (dark green), XBB.1.5 (light green), XBB.1.16 (dark blue), and CH.1 (light orange).

Since its emergence in November 2019, the SARS-CoV-2 virus has undergone continuous mutation and global spread. One variant that has garnered significant attention is Omicron, which was first identified in South Africa in November 2021. Omicron exhibits rapid mutation, making it capable of evading neutralizing antibodies and causing reinfection and breakthrough infections in the population. During this period, particular focus has been placed on the main mutants of Omicron, including BA.5, BF.7, BQ.1, BQ.1.1, XBB, XBB.1, XBB.1.6, and XBB.1.16. The protective efficiency of existing vaccines against mutant strains is shown in **Table 1**. Here, we constructed a radial-loop SARS-CoV-2 sequence by collecting the Global Initiative on Sharing All Influenza Data (GISAID) EpiCoV data using a subsampling technique. We utilized all variant samples available on GISAID as the baseline for targeted phylogenetic analysis (**Figure 3A**) and identified the lineages of the aforementioned eight mutant sequences (**Figure 3B**). Additionally, we collected the critical mutations of these eight mutants within and outside the RBD region of the spike protein (**Figure 3C**).

**Table 1:** The vaccine effectiveness (VE) of various types of vaccines on different mutants.

	BA.5	BF.7	XBB	XBB.1	XBB.1.5	BQ.1	BQ.1.1	XBB.1.16
**Recombinant Protein Vaccines**								
Three doses of vaccine (ZF2001*3)	-7.19 times (pVNT50, PT, pVNT, 1302 vs. 181) (43)	-20.7times (pVNT50, PT, pVNT, 1302 vs. 63) (43)	-217 times (pVNT50, PT, pVNT, 1302 vs. 6) (43)			-144times (pVNT50, PT, pVNT, 1302 vs. 9) (43)	-217times (pVNT50, PT, pVNT, 1302 vs. 6) (43)	
Three doses of vaccine (Recombinant Protein Vaccines *2 +ZF2001 *1)	-30.07 times (pVNT50, PT, pVNT, 812 vs. 27) (43)	-36.90 times (pVNT50, PT, pVNT, 812 vs. 22) (43)	-135.33 times (pVNT50, PT, pVNT, 812 vs. 6) (43)			-135.33times (pVNT50, PT, pVNT, 812 vs. 6) (43)	-135.33times (pVNT50, PT, pVNT, 812 vs. 6) (43)	
**Inactivated Vaccines**								
Three doses of vaccine (Inactivated Vaccines*3)	-5.0 times (pVNT50, PT, pVNT, 131 vs. 26) (43)	-13.1times (pVNT50, PT, pVNT, 131 vs. 10)[1]	-26.2times (pVNT50, PT, pVNT, 131 vs. 5) (43)			-14.56times (pVNT50, PT, pVNT, 131 vs. 9) (43)	-21.83times (pVNT50, PT, pVNT, 13 1vs. 6) (43)	
Three doses of vaccine (CoronaVac [Sinovac] or BBIBP-CorV [Sinopharm])*3	-2.04times (NT50, G614, pVNT, 1-5month, 47 vs. 23) (63)	-3.13times (pVNT50, G614, NT, 1-5month 47 vs. 15) (63)		-7.83times (pVNT50, G614, NT, 1-5month, 47 vs. 6) (63)			-5.88times (pVNT50, G614, NT, 1-5month, 47 vs. 8) (63)	
Three doses of vaccine (CoronaVac*)3	-3.03 times (pVNT50, WT, pVNT, 97 vs. 32) (9)	-3.23 times (pVNT50, WT, pVNT, 97 vs. 30) (9)	-3.23 times (pVNT50, WT, pVNT, 97 vs. 30) (9)	-29times (ID50, D614G, pVNT, 14d, 516 vs. 18) (64)	-3.23 times (pVNT50, WT, pVNT, 97 vs. 30) (9)	-3.23 times (pVNT50, WT, pVNT, 97 vs. 30) (9)	-3.23 times (pVNT50, WT, pVNT, 97 vs. 30) (9)	
	-16times (ID50, D614G, pVNT, 14d, 516 vs. 32) (64)	-15times (ID50, D614G, pVNT, 14d, 516 vs. 35) (64)			-40imes (ID50, D614G, pVNT, 14d, 516 vs. 13) (64)	-29times (ID50, D614G, pVNT, 14d, 516 vs. 18) (64)	-27times (ID50, D614G, pVNT, 14d, 516 vs. 19) (64)	
**mRNA Vaccines**								
Three doses of vaccine (BNT162b2 *3)	-9.45 times (pVNT50, WT, pVNT, 2014 vs. 213) (9) -11.44times(ID50, WA1, pVNT, 2882 vs. 252) (68)	-24.7 times(pVNT50, WT, pVNT, 2014 vs. 83) (9)	-62.94 times (pVNT50, WT, pVNT, 2014 vs. 32) (9)	-435.19times (WA1/2020, pVNT, 2021, 45695 vs. 105) (67)	-57.54 times (pVNT50, WT, pVNT, 2014 vs. 35) (9)	-38.73 times (pVNT50, WT, pVNT, 2014 vs. 52) (9)	-44.75 times (pVNT50, WT, pVNT, 2014 vs. 45) (9) -39.48times (ID50, WA1, pVNT, 2882 vs. 73) (68)	
	-51.52times (WA1/2020, pVNT, 2021, 45695 vs. 887) (67)	-76.80times (WA1/2020, pVNT, 2021, 4569 5 vs. 59) (67)	-80.10times (ID50, WA1, pVNT, 2882 vs. 36) (68)		-90.06times (ID50, WA1, pVNT, 2882 vs. 32) (68)		-175.08times (WA1/2020, pVNT, 2021, 45695 vs. 261) (67)	
Two doses of vaccine (Monovalent mRNA Vaccines*2)	83%(14-150days, during BA.4/BA.5 period) (65) 37%(≥150days, duringBA.4/BA.5 period) (65)							
Three doses of vaccine (Monovalent mRNAVaccines*3)	-12times (ID50, D614G, pVNT, 7687 vs. 628) (14)		<-70times (ID50, D614G, pVNT, 7687 vs. <111) (14)	<-71times (ID50, D614G, pVNT, 7687 vs. <108) (14) -50.36times(pVNT50, G614, pVNT, 1-5month, 1662 vs. 33) (63)		<-37times (ID50, D614G, pVNT, 7687 vs. <208) (14)	<-55times (ID50, D614G, pVNT, 7687 vs. <139) (14)	
	60%(7-120days, duringBA.4/BA.5 period) (65) 29%(≥120days, duringBA.4/BA.5 period) (65)							
	+1.61times (pVNT50, BA.1, pVNT, 1686 vs. 1049, for men) (80)							
	-3.11times (pVNT50, G614, pVNT, 1-5month, 1662 vs. 535) (63)	-5.91times (pVNT50, G614, pVNT, 1-5month, 1662 vs. 281) (63)					-17.68times (pVNT50, G614, pVNT, 1-5month, 1662 vs. 94) (63)	
	-12.66times (FRNT50, UT-NC002-1T, FRNT, 180-189days, 257 vs. 20.3) (66)		-21.60 times (FRNT50, UT-NC002-1T, FRNT, 180-189days, 257 vs. 11.9) (66)				-21.07times (FRNT50, UT-NC002-1T, FRNT, 180-189days, 257 vs. 12.2) (66)	
Four doses of vaccine (Monovalent mRNAVaccines*4)	-16.14times (FFRNT50, USA-WA1/2022, FFRNT, 23-94days, 1533 vs. 95) (69)	-22.22times (FFRNT50, USA-WA1/2022, FFRNT, 23-94days, 1533 vs. 69) (69)	<-145times (ID50, D64G, pVNT, 21182 vs. <147) (14)	-102.2times (FFRNT50, USA-WA1/2022, FFRNT, 23-94days, 1533 vs. 15) (69)		<-43times (ID50, D64G, pVNT, 21182 vs. <496) (69)	-69.68times (FFRNT50, USA-WA1/2022, FFRNT, 23-94days, 1533 vs. 22) (69)	
	61%(7-120days, duringBA.4/BA.5 period) (65)							
	-11.69times (FRNT50, UT-NC002-1T, FRNT, 33-57days, 727 vs. 62.2) (66)		-108.30times (FRNT40, UT-NC002-1T, FRNT, 1527 vs. 14.1) (11)		-114.81times (FRNT40, UT-NC002-1T, FRNT, 1527 vs. 13.3) (11)		-43.27times (FRNT50, UT-NC002-1T, FRNT, 33-57days, 727 vs. 16.8) (66)	
	-14times (ID50, D64G, pVNT, 21182 vs. <1540) (14)		-51.56times (FRNT50, UT-NC002-1T, FRNT, 33-57days, 727 vs. 14.1) (66)	<-155times (ID50, D64G, pVNT, 21182 vs. <137) (14)			<-81times (ID50, D64G, pVNT, 21182 vs. <261) (14)	
Four doses of vaccine (BNT162b2 *3+Bivalent vaccine Booster *1)	-4.32times (FRNT50, USA-WA1/2020, Participants without SARS-CoV-2 Infection before Dose 4, 2237 vs. 518) (70)		-56.75times (ID50, WA1, pVNT, 11009 vs. 194) (68)	-40.67times (FRNT50, USA-WA1/2020, Participants without SARS-CoV-2 Infection before Dose 4, 2237 vs. 55) (70)	-54.23times (ID50, WA1, pVNT, 11009 vs. 203) (68)		-15.64times (FRNT50, USA-WA1/2020, Participants without SARS-CoV-2 Infection before Dose 4, 2237 vs. 143) (70)	
	-3.52times (FRNT50, USA-WA1/2020, Participants with SARS-CoV-2 Infection before Dose 4, 4847 vs. 1377) (70)			-37times (FRNT50, USA-WA1/2020, Participants with SARS-CoV-2 Infection before Dose 4, 4847 vs. 131) (70)			-10.92times (FRNT50, USA-WA1/2020, Participants with SARS-CoV-2 Infection before Dose 4, 4847 vs. 444) (70)	
	-4.35times (ID50, WA1, pVNT, 11009 vs. 2533) (68)						-27.52times (ID50, WA1, pVNT, 11009 vs. 400) (68)	
Four doses of vaccine (BNT162b2 *3+Monovalent vaccine Booster *1)	-14.89times (FRNT50, USA-WA1/2020, Participants without SARS-CoV-2 Infection before Dose 4, 1325 vs. 89) (70)			-77.94times (FRNT50, USA-WA1/2020, Participants without SARS-CoV-2 Infection before Dose 4, 1325 vs. 17) (70)			-53times (FRNT50, USA-WA1/2020, Participants without SARS-CoV-2 Infection before Dose 4, 1325 vs. 25) (70)	
	-8.14times (FRNT50, USA-WA1/2020, Participants with SARS-CoV-2 Infection before Dose 4, 5120 vs. 629) (70)			-52.24times (FRNT50, USA-WA1/2020, Participants with SARS-CoV-2 Infection before Dose 4, 5120 vs. 98) (70)			-38.79times (FRNT50, USA-WA1/2020, Participants with SARS-CoV-2 Infection before Dose 4, 5120 vs. 132) (70)	
Four doses of vaccine (monovalent BNT162b2/Comirnaty vaccine booster)*1					-151.33 times (PVNT50, B.1, PVNT, 454 vs. 3) (75)			-113.50 times (PVNT50, B.1, PVNT, 454 vs. 4) (75)
Four doses of vaccine (bivalent BNT162b2/Comirnaty Original/Omicron BA.4-5 vaccine booster)*1					-44.27 times (PVNT50, B.1, PVNT, 974 vs. 22) (75)			-57.29times (PVNT50, B.1, PVNT, 974 vs. 17) (75)
Monovalent mRNA Booster*1 (most of whom had received three previous doses of vaccine.)	-7.60times (WA1/2020, pVNT, 2022, 21507 vs. 2276) (67)	-9.45times (WA1/2020, pVNT, 2022, 21507 vs. 2829) (67)		-126.51times (WA1/2020, pVNT, 2022, 21507 vs. 170) (67)			-52.97times (WA1/2020, pVNT, 2022, 21507 vs. 406) (67)	
Bivalent mRNA Booster*1 (most of whom had received three previous doses of vaccine.)	-10.97times (WA1/2020, pVNT, 2022, 40515 vs. 3693) (67)	-16.89times (WA1/2020, pVNT, 2022, 40515 vs. 2399) (67)		-231.51times (WA1/2020, pVNT, 2022, 40515 vs. 175) (67)			-79.75times (WA1/2020, pVNT, 2022, 40515 vs. 508) (67)	
Monovalent mRNA Booster*1	-17.14times (FRNT50, WA1/2020, FRNT, 857 vs. 50) (21)						-45.11times (FRNT50, WA1/2020, FRNT, 857 vs. 19) (21)	
Monovalent mRNA Booster*2	-9.41times (FRNT50, WA1/2020, FRNT, 2352 vs. 250)^21^		-63.57times (FRNT50, WA1/2020, FRNT, 2352 vs. 63.6) (21)				-32.22times (FRNT50, WA1/2020, FRNT, 2352 vs. 32.2) (21)	
Bivalent mRNA Booster*1	-4.31times (FRNT50, WA1/2020, FRNT, 2481 vs. 576) (21)		-25.84times (FRNT50, WA1/2020, FRNT, 2482 vs. 96) (21)				-22.15times (FRNT50, WA1/2020, FRNT, 2482 vs. 112) (21)	
Four doses of vaccine (bivalent (WT and BA.5) COVID-19 mRNA vaccines booster)*1	<-8.1times (ID50, D614G, pVNT, 13736 vs. 1688) (14)		<-66times (ID50, D614G, pVNT, 13736 vs. <209) (14)	<-85times (ID50, D614G, pVNT, 13736 vs. <162) (14)		<-24times (ID50, D614G, pVNT, 13736 vs. <568) (14)	<-41times (ID50, D614G, pVNT, 13736 vs. <337) (14)	
Five doses of vaccine (monovalent mRNA : BNT162b2 Or mRNA1273)*4+ (Bivalent(ancestral+BA.4/5)mRNA vaccine)*1			-36.57imes (FRNT40, UT-NC002-1T, FRNT, 1086 vs. 29.7) (11)		-33.62times (FRNT40, UT-NC002-1T, FRNT, 1086 vs. 32.2) (11)			
BA.5 bivalent booster*1 (Monovalent mRNA *two or three or four before booster)	-12.15times (FFRNT50, USA-WA1/2022, FFRNT, 23-94days, without infection history, 3620 vs. 298) (69)	-11.87times (FFRNT50, USA-WA1/2022, FFRNT, 23-94days, without infection history, 3620 vs. 305) (69)		-103.42times (FFRNT50, USA-WA1/2022, FFRNT, 23-94days, without infection history, 3620 vs. 35) (69)			-49.59times (FFRNT50, USA-WA1/2022, FFRNT, 23-94days, without infection history, 3620 vs. 73) (69)	
	-3.71times (FFRNT50, USA-WA1/2022, FFRNT, 23-94days, with infection history, 5776 vs. 1558) (69)	-4.72times (FFRNT50, USA-WA1/2022, FFRNT, 23-94days, with infection history, 5776 vs. 1223) (69)		-56.08times (FFRNT50, USA-WA1/2022, FFRNT, 23-94days, with infection history, 5776 vs. 103) (69)			-21.63times (FFRNT50, USA-WA1/2022, FFRNT, 23-94days, with infection history, 5776 vs. 267) (69)	
Two, three or four doses of vaccine (monovalent mRNA : BNT162b2 or mRNA-1273 or NA vaccines)*two or three or four +BA.2 breakthrough	-4.80times (NT50, B.1.1, pVNT, 1918 vs. 400) (12)			-15.98times (NT50, B.1.1, pVNT, 1918 vs. 120) (12)	-15.22times (NT50, B.1.1, pVNT, 1918 vs. 126) (12)			-13.50times (NT50, B.1.1, pVNT, 1918 vs. 142) (12)
Two, three or four doses of vaccine (monovalent mRNA : BNT162b2 or mRNA-1273 or NA vaccines) *two or three or four +BA.5breakthrought	-2.51times (NT50, B.1.1, pVNT, 6900 vs. 2741) (12)			-30.67times (NT50, B.1.1, pVNT, 6900 vs. 225) (12)	-37.10times (NT50, B.1.1, pVNT, 6900 vs. 186) (12)			-27.38times (NT50, B.1.1, pVNT, 6900 vs. 252) (12)

*One dose: The vaccine was immunized once. Two doses: the vaccine was immunized twice. Three doses: the vaccine was immunized thrice. Four doses: the vaccine was immunized four times. Booster dose, vaccines added to strengthen immunity. IC50, 50% true virus neutralization titer; ID50, Infection dose 50. pVNT50, 50% pseudovirus neutralization titer. PRNT50, 50% plaque reduction neutralization test. D614G, taking the D614 G mutant as the reference object. WT, taking the wild-type (Wuhan-hu-1), SARS-CoV-2 prototype (PT), UT-NC002-1T, and B.1.1 as the reference. The new variant strain’s GMT compared with the comparative strain’s GMT, with a ratio greater than 0 and a positive multiple. Greater than 0 times (green), 0–10 times (yellow), -10–50 times (blue), -50–100 times (purple), greater than -100 times (red), vaccine effectiveness (orange). 1–5 month: 1–5 months after administration, 33–57 days: 33–57 days after administration, 23–94: 23–94 days after administration.

## BA.5

BA.5 is believed to have originated in early January 2022 (December 10, 2021 - February 6, 2022) (81). BA.5 has spread rapidly around the world. As of August 2022, BA.5 accounted for 75–95% of cases in most countries. BA.5 has a unique Omicron lineage. The ancestral amino acids at 69-70del, L452R, F486V, and position Q493 differ from BA.2 (81). The spike protein of BA.5 contains a unique amino acid substitution, which not only facilitates the evasion of nAbs produced by vaccination but also enhances its binding affinity to the ACE2 receptor (82).

The ZF 2001 protein subunit vaccine was developed by Anhui Zhifei Longkang, who demonstrated that by administering three doses of the homologous ZF 2001 vaccine (ZF 2001 group, 16 subjects), the nAb titer against the BA.5 mutant strain was 7.19 times lower than that of the SARS-CoV-2 prototype (PT) (1302 vs. 181) at a 50% pseudovirus neutralization titer (61). In the same set of experiments, another 16 subjects were vaccinated with three doses of an inactivated vaccine. In the comparison of BA.5 with the PT, the GMT was 5.0 times lower (131 vs. 26) (61). Furthermore, three doses of ZF2001 induced higher nAb titers against PT isolates and BA.5 compared with three doses of the inactivated vaccine.

In an experiment utilizing enzyme-linked immunosorbent assay (ELISA) and lentivirus-based pseudovirus neutralization assays, plasma samples were collected from healthy volunteers within 1–5 months after inoculation with a three-dose inactivated vaccine (Corona Vac [Sinovac [Sinopharm]). The results showed a 2.04 times decrease in neutralization titer against the G614 strain (47 *vs.* 23) (83). In another study, in which individuals were inoculated with three doses of the inactivated vaccine Corona Vac (n = 20), plasma samples were obtained 4–5 weeks after injection, and their neutralization activity was determined. The findings revealed that the geometric mean relative pseudotyped virus neutralization titer 50 (pVNT50) titer of human serum receiving three doses of the Corona Vac vaccine was 3.03 times lower than that of the wild-type (WT) strain (97 vs. 32) (19). Samples collected within 14 days after the third dose of the Corona Vac vaccine exhibited a geometric mean 50% inhibitory dose (ID50) that was 16 times lower than that of the WT strain (516 vs. 32) (84). In addition, relevant studies have shown that BA.5 has more pairs of mutations in the RBD, leading to increased immune escape (85). Therefore, these three doses of inactivated vaccine producing fewer neutralizing antibodies than the original virus strain results may be related to point mutation.

In a study on mRNA vaccines, researchers administered two, three, and four doses of mRNA vaccines to adults aged 18 and above. Different doses of monovalent mRNA vaccines were inoculated, and the effectiveness of these vaccines was evaluated by comparing them with hospitalized individuals who were not vaccinated against COVID-19. During the BA.4/BA.5 outbreaks, the vaccine effectiveness (VE) was 83% at 14–150 days after the second dose and decreased to 37% 150 days later. Similarly, the VE was 60% at 7–120 days after the third dose, decreased to 29% at 120 days, and was 61% at 7–120 days after the fourth dose (86).

Compared with that of D614G, the ID50 titer of BA.4/5 decreased 12-fold (7687 vs. 628) after three doses of the original COVID-19 mRNA vaccine (25). The study observed that the 50% plaque reduction neutralization test (PRNT50) titer in male serum was 1.61 times higher than that of BA.1 (1686 vs. 1049) after two doses of mRNA vaccine and one dose of BNT162b2 vaccine (82). Neutralization assays were conducted on plasma samples obtained from the inoculated individuals at 1–5 months after receiving three doses of an mRNA vaccine (BNT162b2 or mRNA-1273). It was discovered that the pVNT50 titer of human serum receiving three doses of BNT162b2 or mRNA-1273 vaccine was 3.11 times lower than that of G614 (1662 vs. 535) (83). In the same study, individuals who also received three doses of BNT162b2 or mRNA-1273 vaccine demonstrated a 12.66 times lower 50% focus reduction neutralization test (FRNT50) in adult serum collected within 180–189 days after the third dose compared with UT-NC002-1T (257 vs. 20.3) (87).

After receiving three doses of the BNT162b2 vaccine (Pfizer-BioNTech), serum samples were collected 20 days (15–28 days) after the third injection dose. The Luciferase pseudovirus neutralization test was used for determination. The nAb titer of BA.5 was found to be 51.52 times lower (45695 vs. 887) than that of WA1/2020. In another two studies involving three doses of the BNT162b2 vaccine, one neutralization assay was conducted on plasma samples obtained from vaccinated individuals at 4–5 weeks post-vaccination. The results showed that the geometric mean pVNT50 titer was 9.45 (2014 vs. 213) times lower than that of the WT (88). Additionally, in another study using three doses of the BNT162b2 vaccine, the ID50 value for BA.5 was 11.44 times lower (2882 vs. 252) than that for WA1 (89).

After four doses of mRNA-1273 or BNT162b2, the GMT of serum collected from individuals at 23–94 days (median 47 days) after injection was 16.14 times lower than that of USA–WA1/2020 (1533 vs. 95) (90). Compared with D614G, the ID50 titer of BA.4/5 decreased by 14 times (21182 vs. < 1540) after four doses of the original COVID-19 mRNA vaccine (25). Another study administered participants with four doses of BNT162b2 or mRNA-1273 and collected adult serum within 33–57 days after the fourth injection dose. The FRNT50 was 11.69 times lower than that of UT-NC002-1T (727 vs. 62.2) (87). The sampling time of serum samples for the third and fourth doses of the mRNA vaccine was different, and the data also showed a decrease in anti-spiking immunity over time.

In a comparative study of monovalent and bivalent vaccines, after the fourth dose of BNT 162 b2 booster vaccine, the authors compared BNT 162 b2 with USA-WA1/2020 using the FRNT50 for BA.5. The GMTs of participants who were not infected with SARS-CoV-2 before the fourth dose, infected with SARS-CoV-2, and vaccinated with monovalent booster (BNT 162 b2) were 14.89 (1325 vs. 89) and 8.14 (5120 vs. 629) times lower than that of USA-WA1/202. In the group who received four vaccinations with bivalent vaccine (BNT 162 b2 bivalent vaccine, Pfizer-BioNTech), the uninfected group and the infected group had titers that were 4.32 (2237 *vs.* 518) and 3.52 (4847 vs. 1377) times lower, respectively, compared with that of wild-type D614G (91). The vaccine was spiked with BA.4/5 and wild type (D614G). The neutralization titer ratio of the bivalent vaccine to the monovalent vaccine was 5.8 in the uninfected group and 2.7 in the infected group. These findings suggest that the bivalent vaccine is more immunogenic than the original vaccine. In another study, the nAb titer of the BA.5 mutant was 7.60 (21507 vs. 2276) and 10.97 (40515 vs. 3693) times lower than that of the WA1/2020 strain after receiving monovalent or bivalent mRNA enhancers, respectively (88). In contrast, we administered one dose of monovalent RNA enhancer (n = 12, d7–28), two doses of monovalent mRNA enhancer (n = 11, d6–57), or one dose of bivalent RNA enhancer (n = 12, d16–42). By comparing the FRNT 50 of the nAbs against the multi-particle subvariety, the neutralizing titers were 17.14, 9.41, and 4.31 times lower than that of WA 1/2020 (857 vs. 50, 2352 vs. 250, and 2481 vs. 576) (33). The serological data showed that for the BA.5 mutant, one dose of bivalent mRNA enhancer was better than two doses of monovalent mRNA enhancer.

In another bivalent mRNA vaccine study (WT and BA.5 COVID-19 mRNA vaccine booster), participants received three doses of the original COVID-19 mRNA vaccine before receiving a fourth vaccination. The study found that for BA.5 compared to D614G, the individual neutralization ability decreased by 8.1 times (13736 vs. 1688), resulting in a higher nAb titer (92). The bivalent vaccine contains the spike proteins of WA.1 and BA.5, which improved the neutralization of BA.5 compared with that of the monovalent vaccine. Participants in the BA.5 bivalent booster vaccination study received two, three, or four doses of the parental monovalent mRNA vaccine before the BA.5 bivalent booster vaccination. The data showed that the GMTs of the previously uninfected SARS-CoV-2 group and the infected SARS–CoV-2 group were 12.15 times lower (3620 vs. 298) and 3.71 times lower (5776 vs. 1558), respectively, compared to the USA-WA1/2020 after vaccination with the bivalent mRNA vaccine booster (90). Overall, bivalent vaccines produce higher nAb titers than monovalent vaccines, highlighting the need to develop newer bivalent mRNA boosters to enhance human immunity.

## BF.7

BF.7 is derived from the Omicron BA.5 mutant, also referred to as BA.5.2.1.7, which is a variant of Omicron. The variant was initially identified in Belgium on May 13, 2022 (93) and carried an additional R346T mutation in the receptor-binding region of the RBD compared with BA.4/5. This spike protein mutation confers increased immune escape ability and a higher transmission rate (94).

In a study, Li et al. compared the neutralizing activity of three doses of homologous ZF 2001 protein subunit vaccine (ZF 2001 group, 16 subjects) and three doses of inactivated vaccine against Omicron BF.7. The GMTs of the BF.7 mutant were 20.07 (1302 vs. 63) times and 13.10 (131 vs. 10) times lower than that of the PT in the pseudovirus neutralization test (61). In this experiment, vaccination with three doses of ZF 2001 was found to be superior to vaccination with three doses of the inactivated vaccine for BF.7. In addition, in the same group of experiments, the GMTs of BA.5 after vaccination with ZF2001 (three doses) and inactivated vaccine (three doses) were 181 and 26, respectively. Compared with BA.5, the GMTs of BF.7 (63 and 10 in the two groups, respectively) decreased by 2.87 (181 vs. 62) and 2.6 (26 vs. 10) times, respectively. This also indicated that BA.7 has a more robust immune escape ability than BA.5.

In another experiment involving the injection of an inactivated vaccine, plasma samples were collected from healthy volunteers at 1–5 months after receiving three doses of either CoronaVac (Sinovac) or BBIBP-CorV (Sinopharm) inactivated vaccines. The neutralization titer of the vaccine, as measured by ELISA and lentivirus-based pseudovirus neutralization assays, was found to be 3.13 times (47 *vs.* 15) lower than that of the G614 strain (83). In another study, plasma samples were obtained from individuals receiving CoronaVac (three doses) at 4–5 weeks after vaccination, and the neutralization efficiency was 3.23 (97 vs. 30) times lower than that of the WT (19). Moreover, the participants received three doses of the CoronaVac vaccine, and the ID 50 of adult serum collected within 14 days after the third dose of CoronaVac was 15 (516 vs. 35) times lower than that of the WT (D614G) (92). In the four studies involving three doses of the vaccine, the vaccine-induced nAbs were found to be less effective.

In an mRNA vaccine study, at 4–5 weeks after vaccination with the BNT 162 b2 vaccine (three doses), neutralization assays were performed from plasma samples obtained from the participants. The geometric mean pVNT50 titer of the serum of those who receiving three doses of the BNT 162 b2 vaccine was found to be 24.7 (2014 vs. 83) times lower than that of the WT (19). Similarly, after receiving BNT 162 b2 (Pfizer-BioNTech, three doses), Miller et al. used the pseudovirus neutralization test of luciferase and determined that the nAb titers of BF.7 and WA1/2020 were 76.80 (45695 vs. 595) times lower than those of the WT (19). In Fang’s study, in the clinical data of BNT162b2or mRNA-1273 (three doses), the neutralization titer of pVNT50 was 5.91 (1662 vs. 281) times lower than that of G614 according to the pseudovirus neutralization assay (83). In another study, participants were vaccinated with four doses of mRNA-1273 or BNT162b2, and the GMT of serum collected from individuals at 23–94 days (median 47 days) after vaccination was 22.22 times lower than that of USA-WA1/2020 (1533 vs. 69) (90). Whether after three doses or four doses of mRNA vaccine, the number of antibodies produced was higher than that induced by three doses of ZF2001 or three doses of inactivated vaccine.

In the study of monovalent and bivalent mRNA vaccines against BF.7, one study measured the neutralizing antibody titers of participants (most of them had already received three doses of vaccines) after receiving monovalent mRNA enhancers and receiving bivalent mRNA enhancers in 2022. The results showed that the neutralizing titers against BF.7 mutation were 9.45 (21507 vs. 2829) and 16.89 (40515 vs. 2399) times lower than those against WA1/2020 in individuals inoculated with monovalent and bivalent mRNA enhancers, respectively (88). The titers of neutralizing antibodies produced by BA.5 in this group of experiments were 2276 (monovalent mRNA booster) and 3693 (bivalent mRNA booster), respectively. In another study, participants were first vaccinated with two, three, or four doses of parental monovalent mRNA vaccine, inoculated with BA.5 bivalent enhancer, and then compared with USA-WA1/2020. The GMTs of the previously uninfected SARS-CoV-2 and the infected SARS-CoV-2 groups were 11.87 (3620 vs. 305) and 4.72 (5776 vs. 1223) times lower than that of USA-WA1/2020, respectively (90). The titers of neutralizing antibodies produced by BA.5 in this group of experiments were 298 (uninfected group) and 1558 (infected group), respectively. In these two experiments, the results showed that bivalent mRNA vaccination was superior to monovalent mRNA vaccination. In this study of the bivalent vaccine over two experiments, the deviation of the geometric mean and neutralizing antibody titer of serum samples infected with OmicronBA.5 and OmicronBF.7 was no more than 1.54 times (3693 vs. 2399, 305 vs. 298, 1558 vs. 1223), respectively. Thus, the bivalent mRNA vaccine induced a stronger and broader antibody response than the monovalent mRNA vaccine.

## BQ

BQ.1 was first discovered in Nigeria in June 2022, while BQ.1.1, as the first sub-branch of BQ.1, rapidly spread to Europe and North America with BQ.1. As of November 19, 2022, BQ.1 and BQ.1.1 accounted for 25.5% and 24.2% of the total cases, respectively (25, 90). Compared with BA.5, the BQ.1 variant exhibited two mutations in the spike protein: K444T and N460K. The BQ.1.1 variant also possessed three mutations in the spike protein: K444T, N460K, and R346T. These mutations confer a high level of infectivity by evading immunity (95).

For a recombinant protein and inactivated vaccine, in the group inoculated with the ZF2001 protein subunit vaccine (three doses, n = 16) and the group inoculated with three doses of inactivated vaccine (three doses, n = 16), the GMTs of BQ.1 and BQ.1.1 at 50% pseudovirus neutralization titers were 144 (1302 vs. 9) and 217 (1302 vs. 6) times lower than that of the PT, respectively. Serum positivity (defined as neutralization titer > 10 [detection limit]) was 44% in the ZF2001 group but was only 13% in the BQ.1.1 group. It decreased by 14.56 (131 vs. 9) and 21.38 (131 vs. 6) times in the inactivated vaccine group, respectively (61). In another three studies, the first study administered three doses of inactivated vaccine (CoronaVac [Sinovac] or BBIBP-CorV [Sinopharm]), and plasma samples were collected from healthy volunteers within 1*–*5 months after vaccination. ELISA and lentivirus-based pseudovirus neutralization assays showed that the neutralization titer of BQ.1.1 was 5.88 (47 vs. 8) times lower than that of G614 strain (83). In the second study, which administered three doses of CoronaVac vaccine (Beijing Kexing Zhongwei Biotechnology Co., Ltd.), the results showed that the pVNT50 values for BQ.1 and BQ.1.1 mutants were 3.23 (97 vs. 30) times lower than that of the WT (19). In the third study, serum samples were collected within 14 days after the third dose of CoronaVac vaccine. The ID50 values of BQ.1 and BQ.1.1 mutants were 29 (516 vs. 18) and 27 (516 vs. 19) times lower than the ID50 values for D614G, respectively (84). Based on the above data, we deduced that after three doses of ZF2001 or three doses of inactivated vaccine, the number of neutralizing antibodies against BQ.1 and BQ.1.1 is limited, and the neutralizing activity is reduced, indicating that the two mutants have strong escape ability.

In a study of three doses of an mRNA vaccine, after the first dose of the original COVID-19 mRNA vaccine, the individual’s neutralization was approximately 37 (7687 vs. < 208) and 55 (7687 vs. < 139) times lower than the ID50 of D614G for BQ.1 and BQ.1.1, respectively (25). In a second study, a neutralization assay was performed 1–5 months after three doses of an mRNA vaccine (BNT162b2 or mRNA-1273). It was found that the geometric mean pVNT50 titer in the serum of BQ.1.1 was 17.68 (1662 vs. 94) times lower than that of G614. A third study showed that the FRNT50 of BQ.1.1 was 21.07 (257 vs. 12.2) times lower than that of UT-NC002-1T when adult serum was collected within 180–189 days after the third dose (87). In a study of the same three doses of the BNT 162 b2 vaccine, a plasma sample obtained from a vaccinated person at 4–5 weeks after vaccination was used for a neutralization test. The pVNT50s of BQ.1 and BQ.1.1 were 38.73 (2014 vs. 52) and 44.75 (2014 vs. 45) times lower than the WT, respectively (87). Another study showed that the ID50 of BQ.1.1 was 39.48 (2882 vs. 73) times lower than that of WA1 (89).

The luciferase pseudovirus neutralization test determined the neutralizing antibody titer of BQ.1.1 as 175.08 (45695 vs. 261) times lower than that of WA1/2020 (88). Individuals who received three doses of the mRNA vaccine had significantly higher GMTs against BQ.1 and BQ.1.1 compared with those who received three doses of recombinant protein vaccine or three doses of inactivated vaccine. However, it is noteworthy that antibodies against BQ decreased over time, raising concerns about vaccine efficacy. These concerns have prompted support for the development of a new vaccine with long-term protection.

The ID50 titers of BQ.1 and BQ.1.1 were 43 (21182 vs. < 496) and 81 (21182 vs. < 261) times lower than that of D614G, respectively, after four doses of the original COVID-19 mRNA vaccine (25). In another study, participants received four doses of BNT162b2 or mRNA-1273, and adult serum was collected within 33–57 days after the fourth dose of injection. The FRNT50 of BQ.1.1 was 43.27 (727 *vs.* 16.8) times lower than that of UT–NC002-1T (87). Compared with three doses of mRNA vaccine, four doses could induce higher titers. Still, the number of antibodies decreased over time and did not cause strong neutralization of BQ.1 and BQ.1.1. Therefore, we also need to develop a new vaccine to deal with mutant strains with a strong escape ability.

In another study of mRNA vaccines, a bivalent mRNA vaccine was administered after receiving three doses of a monovalent BNT 162 b2 mRNA vaccine (Pfizer/BioNTech, n = 21). The neutralization ability of individuals inoculated with bivalent mRNA BQ.1.1 was 27.52 (11009 vs. 400) times lower than that of WA1 (89). In a study of BQ.1 and BQ.1.1, three doses of the original COVID-19 mRNA vaccine were injected first, and then the fourth injection (WT and BA.5) of the COVID-19 mRNA vaccine booster was performed. It was found that compared with D614G, the individual neutralization ability decreased by 24 (13736 vs. < 568) and 41 (13736 vs. < 337) times, respectively (25). In a BA.5 bivalent booster vaccination study, participants received two, three, or four doses of parental monovalent mRNA vaccine before BA.5 bivalent booster vaccination. The data showed that the FFRNT50 values of BQ.1.1 in the previously uninfected SARS-CoV-2 group and the infected SARS-CoV-2 group were 49.59 (3620 vs. 73) times and 21.63 (5776 vs. 267) times lower, respectively, than that of USA-WA1/2020 after inoculation with BA.5 (90). Individuals with SARS-CoV-2 produced more and more extensive neutralization of BQ.1.1 after BA.5 bivalent enhancers. In general, the number of antibodies induced against BQ.1 and BQ.1.1 is small after vaccination with recombinant protein and inactivated vaccines, although the neutralization effect can be enhanced by vaccination with bivalent enhancers.

## XBB

XBB is a recombination of BA.2.10.1 and BA.2.75. XBB was first discovered in India in August 2022 (90), and subsequently derived XBB.1, XBB.1.5, and XBB.1.16 variants were detected. Compared with BA.2, XBB contains five additional mutations in the N-terminal domain (NTD) and nine additional mutations in the RBD (25). XBB.1 and XBB.1.5, in addition to the mutations present in XBB, also carry a key mutation, F486P. This F486P mutation is also found in XBB.1.16. Moreover, XBB.1.16 has an additional E180V mutation in the NTD and an additional T478R mutation in the RBD compared with XBB.1.5 (22). As the number of mutations increases, the mutant strain’s ability to evade the immune response also increases.

In a recombinant protein vaccine, three doses of the ZF 2001 white subunit vaccine (ZF 2001 group, 16 subjects) were produced by Anhui Zhifei Longkang. The neutralizing antibody titer of the XBB mutant in the pseudovirus neutralization experiment was 217 times lower than that of the PT (1302 vs. 6) (61). In this experiment, it was assumed that a neutralization titer higher than 10 indicated serum positivity, which was only 13% for XBB. In the first study, 16 participants were vaccinated with three doses of inactivated vaccine in the other four groups of inactivated vaccine. In the pseudovirus experiment, the GMT of XBB was 26.20 (131 vs. 5) times lower than that of the PT (43). In a study of XBB.1, participants were vaccinated with three doses of inactivated vaccine (Corona Vac [Sinovac] or BBIBP-Corv [Sinopharm]), and plasma samples were collected from healthy volunteers within 1–5 months after vaccination. ELISA and lentivirus-based pseudovirus neutralization assays showed that the neutralization titer was 7.83 (47 vs. 6) times lower than that of the G614 strain (83). In a third study, plasma samples obtained from vaccinated individuals 4–5 weeks after vaccination were neutralized by a three-dose inactivated vaccine (Corona Vac, n = 20) schedule. It was found that the pVNT50 values of XBB and XBB.1.5 were 3.23 (97 vs. 30) times lower than that of the WT (19). In the last study, which also administered three doses of CoronaVac vaccine, adult serum was collected within 14 days after the third dose. It was found that the ID50 values of XBB.1 and XBB.1.5 were 29 (516 vs. 18) and 40 (516 vs. 13) times lower than that of WT, respectively (84). In the recombinant protein vaccine and inactivated vaccine group, for XBB and XBB-derived new mutants, the new spike mutations led to an increase in antibody evasion, resulting in a very small number of neutralizing antibodies. The neutralization effect of the new mutant strains derived from XBB and XBB was significantly reduced.

In a study administering three doses of BNT 162 b2 vaccine, a neutralization assay was performed on plasma samples obtained from vaccinated individuals at 4–5 weeks after vaccination. For XBB and XBB.1.5, it was found that the geometric mean pVNT50 titer of serum of people receiving three doses of BNT 162 b2 vaccine was 62.94 (2014 vs. 32) and 57.54 (2014 vs. 35) times lower than that of WT, respectively (19). In another study, the ID50 values for XBB and XBB.1.15 were 80.10 (2882 vs. 36) and 90.06 (2882 vs. 32) times lower than the ID50 values of WA1, respectively (89). In a third study, the pseudovirus neutralization test using luciferase was used for determination, and the neutralizing antibody titer of XBB.1 was 435.19 (45695 *vs.* 105) times lower than that of WA1/2020. Compared with D614G, the ID50 titers of XBB and XBB.1 decreased by 70 (7687 vs. < 111) and 71 (7687 vs. < 108) times, respectively, after three doses of the original COVID-19 mRNA vaccine (25). In a study of XBB, three doses of BNT162b2 or mRNA-1273 vaccine were also inoculated. The FRNT50 of adult serum collected within 180–189 days after the third dose was 21.60 (257 *vs.* 11.9) times lower than that of UT-NC002-1T (87). In a study of three doses of mRNA vaccine (BNT162b2 or mRNA-1273), the plasma samples obtained from vaccinated persons were neutralized 1–5 months after vaccination. It was found that after receiving three doses of an mRNA vaccine (BNT162b2 or mRNA-1273) vaccine, the geometric mean pVNT50 titer value of XBB.1 was 50.36 (1662 vs. 33) times lower than that of G614.

In a study of four doses of mRNA vaccine inoculation, comprising inoculation with the first four doses of the original COVID-19 mRNA vaccine, the ID50 titers of XBB and XBB.1 were lower than 145 (21182 vs. < 147) and less than 155 (21182 vs. < 137) times compared with D614G (25). In the second study of XBB and XBB.1.5, the FRNT50 was determined in Vero E6-TMPRSS 2-T2 A-ACE 2 cells at 33–57 days after injection by inoculating a total dose of four mRNA vaccines (BNT162b2 or mRNA-1273). The GMTs were 108.30 (1527 vs. 14.1) and 114.81 (1527 vs. 13.3) times lower, respectively, than that of the UT–NC002-1T strain. In a third study that also administered four doses of BNT162b2 or mRNA-1273, adult serum was collected within 33–57 days after the fourth dose of injection. The FRNT50 of XBB was 51.56 (727 vs. 14.1) times lower than that of UT–NC002-1T (87). In a fourth study, participants were vaccinated with four doses of the vaccine (mRNA-1273 or BNT162b2). In serum collected from individuals at 23–94 days (median 47 days) after vaccination, the GMT of XBB.1 was 102.2 (1533 vs. 15) times lower than that of USA–WA1/2020 (90).

In a comparative study of monovalent and bivalent mRNA vaccines, the FRNT50 value of XBB.1 was compared with the FRNT50 value of USA-WA1/2020 in the group inoculated with the BNT 162 b2 monovalent booster vaccine as the fourth dose. It was found that the GMT of XBB.1 was 77.94 (1325 vs.17) times lower than that of USA-WA1/2020 in the group not infected with SARS-CoV-2 before the fourth dose and inoculated with a monovalent booster (BNT 162 b2). The GMT of the group infected with SARS-CoV-2 before the fourth dose and inoculated with a monovalent enhancer (BNT 162 b2) was 52.24 (5120 vs. 98) times lower. In the fourth vaccination with the bivalent (BA.4-BA.5) vaccine group, the GMTs of the uninfected group and the infected group were 40.67 (2237 vs. 55) and 37 (4847 vs. 131) times lower, respectively (91). For XBB, the neutralizing rate induced by the bivalent (BA.4-BA.5) vaccine was low, and the GMT ratio of bivalent and monovalent vaccines in the uninfected group was 3.23 (55 vs. 17). The ratio of the infected group was 1.34 (131 vs. 98). This indicates that the BA.4-BA.5 bivalent vaccine has a better and broader neutralization effect than the monovalent vaccine. In another comparative study of two mRNA vaccines, the first one was determined using a pseudovirus neutralization test using luciferase. After receiving monovalent or bivalent mRNA enhancers, the neutralizing antibody titer of the XBB.1 mutant strain was 126.51 (21507 vs. 170) and 231.5 (40515 vs. 175) times lower than that of the WA1/2020 strain (88). The second experiment comprised the study of XBB.1.5 and XBB.1.16. After the injection of a fourth dose of a booster (monovalent BNT162b2/Comirnaty vaccine booster or bivalent BNT162b2/Comirnaty Original/Omicron BA.4-5 vaccine booster), the two groups showed that the neutralization titers of XBB.1.5 and XBB.1.16 were 151.33 (454 vs. 3) and 44.27 (974 vs. 22) times and 113.50 (454 vs. 4) and 57.29 (974 vs. 17) times lower than that of D614G, respectively (96). In the two studies, the ratios of neutralizing antibodies induced by bivalent-to-monovalent XBB.1 were 1.03 (175 *v*s. 170), 7.3 (22 vs. 3) for XBB.1.5 and 4.25 (17 vs. 4) for XBB.1.16.

For another mRNA vaccine study, the participants received two doses of monovalent RNA enhancer (n = 11, d6–57) or one dose of bivalent RNA enhancer (n = 12, d16–42). The FRNT50 values of XBB were 63.57 (2352 vs. 63.6) and 25.84 (2482 vs. 96) times lower, respectively, than those of WA1/2020 by comparing the FRNT50 of neutralizing antibodies against multi-particle subvariants. For XBB, compared with two doses of mRNA vaccine, one dose of bivalent mRNA vaccine was more effective (33).

In the XBB and XBB.1.5 study, participants received three doses of monovalent BNT 162 b2 mRNA (Pfizer/BioNTech, n = 21) followed by one dose of bivalent mRNA vaccine. The neutralization activities of individuals inoculated with bivalent mRNA were 56.75 (11009 vs. 194) and 54.32 (11009 vs. 203) times lower than that of WA1, respectively (89). In the XBB and XBB.1 study, three doses of the original COVID-19 mRNA vaccine were injected before the fourth vaccination (WT and BA.5) of the COVID-19 mRNA vaccine booster. It was found that compared with D614G, the individual neutralization ability decreased by 66 (13736 vs. < 209) and 85 (13736 vs. < 162) times, respectively (25). In the XBB.1 study, participants were first vaccinated with two, three, or four doses of the parental monovalent mRNA vaccine, followed by one dose of the BA.5 bivalent enhancer. The data showed that the GMTs of the previously uninfected SARS-CoV-2 group and the infected SARS-CoV-2 group were 103.42 (3620 vs. 35) and 56.08 (5776 vs. 103) times lower, respectively, than that of the USA-WA1/2020 group after BA.5 bivalent booster inoculation (90). Although the bivalent mRNA vaccine can produce more neutralizing antibodies than the monovalent mRNA vaccine, it still cannot produce potent neutralizing antibodies against XBB and XBB–derived mutants.

Overall, the vaccine’s neutralizing activity against XBB and XBB-derived strains decreased (97), highlighting the importance of developing a new generation of SARS-CoV-2 vaccines based on the development of XBB and XBB-derived pre-mutants or studying heterologous vaccination.

## Effect of heterologous inoculation on the effectiveness of Omicron vaccines

The emergence of new mutants with stronger immune escape ability has increased the demand for booster vaccinations containing updated vaccine components. In contrast to homologous vaccination, combining vaccines from diverse platforms or distinct SARS–CoV–2 strains might elicit a more robust cellular and humoral immune response and achieve broader immunity to newly emerging mutant strains. The protective efficiency of heterologous vaccination of existing vaccines against mutant strains is shown in **Table S2**.

Zhu et al. constructed a pseudo-vesical stomatitis virus panel to evaluate the new mutant strain’s antibody evasion ability. Three groups of experiments were undertaken. The first group was inoculated with two doses of the inactivated vaccine CoronaVac and one dose of the BNT 162b2 vaccine (n = 20). The second group was inoculated with one dose of the aerosol vaccine Ad5-nCoV (n = 20) after two doses of inactivated vaccine CoronaVac. The third group was inoculated with one dose of the aerosol vaccine Ad5-nCoV (n = 20) after three doses of the inactivated vaccine CoronaVac. After 45 weeks of inoculation, the plasma samples obtained from the inoculants were neutralized and determined. The pVNT50s of the first group for BA.5, BF.7, BQ.1, BQ.1.1, XBB, and XBB.1.5 were 11.09, 21.07, 28.36, 34.30, 47.58, and 46.09 times lower than that of the WT (1475 vs. 133, 70, 52, 43, 31, and 32), respectively. The pVNT50s of BA.5, BF.7, BQ.1, BQ.1.1, XBB, and XBB.1.5 in the second group were 8.4, 18.10, 19.56, 26.03, 71.59, and 73.25 times lower than that of the WT (3150 vs. 375, 174, 161, 121, 44, and 43), respectively. The pVNT50s of BA.5, BF.7, BQ.1, BQ.1.1, XBB, and XBB.1.5 in the third group were 4.14, 6.93, 9.82, 12.74, 22.45, and 26.19 times lower than that of the WT (943 vs. 228,136, 96, 74, 42, and 36), respectively (19). Compared to group 1, group 2, which was vaccinated with two doses of the inactivated vaccine CoronaVac followed by one dose of the aerosol vaccine Ad5-nCoV, exhibited higher titers. In the comparison between group 2 and group 3, group 2 induced higher antibody titers than group 3. Schaefer-Babajew et al. demonstrated that pre-existing high–affinity antibodies can hinder immunity by reducing the activation threshold of B cells and directly concealing their homologous epitopes, which might explain the occurrence of the phenomenon observed in group 3 (98). Although the two-dose inactivated vaccine CoronaVac and the one-dose aerosol vaccine Ad5-nCoV outperformed other vaccination strategies, the neutralizing activity of XBB and XBB.1.5 was extremely low in all groups in this study.

Dehesa-Canseco et al. collected data from participants who were vaccinated with a single dose of Ad5-nCoV for eight months and then vaccinated with the mRNA-1273 vaccine booster for three weeks in Emosillo, Sonora, Mexico. Compared with the ancestral strain (B.1.189), the GMTs of BA.5.1.6 and XBB.1 decreased by 3.10 and 34.09 times (221.30 vs. 71.36, 6.49) times, respectively (99). Eight months after receiving a single dose of the Ad5-nCoV vaccine, the GMTs of BA.5.1.6 and XBB.1 were 12.28 and 3.17, respectively. This indicated that vaccination with the mRNA-1273 vaccine increased nAbs, but the numbers of nAbs against XBB.1 were still low.

Hannawi et al. conducted a study to assess the immunogenicity of bivalent and tetravalent protein-enhanced vaccines in male humans. Ninety-four percent of the 450 participants received two doses of the mRNA vaccine. The participants were divided into three groups: Group 1 received one dose of BNT162b2, Group 2 received 20 μg SCTV01C, and Group 3 received 30 μg of SCTV01E. Serum detection was performed 28 days after vaccination. The PRNT50 of BA.5 in group 1 was 1.61 times higher than that of BA.1 (1687 vs. 1049). The PRNT50 of BA.5 in group 2 was 1.46 times higher than that of BA.1 (1736 vs. 1189). In group 3, the PRNT50 of BA.5 was 1.37 times higher than that of BA.1 (2281 vs. 1659) (77). SCTV01E is a tetravalent vaccine formed by the addition of OmicronBA.1 and Delta (B.1.617.2) spike-ECD protein mixtures to the SCTV01C (Alpha + Beta) bivalent vaccine. SCTV01E, as a tetravalent vaccine, induced a higher nAb response to BA.5 than BNT162b2 and SCTV01C. Therefore, tetravalent vaccines might prove to be an effective approach to addressing the challenges posed by the current epidemic.

Lyke et al. conducted a study to evaluate the immunogenicity of the NVX-CoV2373 vaccine against BA.5, BQ.1.1, and XBB.1. The participants were divided into three groups. The first group received either one or two doses of the Ad26.COV2. S vaccine, followed by a single dose of the NVX-CoV2373 vaccine. The second group received two doses of the Moderna mRNA-1273 100-mcg vaccine, followed by a single dose of the NVX-CoV2373 vaccine. The third group received a single dose of the NVX-CoV2373 vaccine after being inoculated with the BioNTech BNT162b2 30-mcg vaccine. The NVX-CoV2373 vaccine is a recombinant nanoparticle vaccine containing 5 micrograms of recombinant spike protein and 50 micrograms of Matrix-adjuvant. At 29 days after vaccination with NVX-CoV2373, the ID50s of BA.5, BQ.1.1, and XBB.1 in group 1 were 5.3, 23, and 61.6 times lower than that of D614G (499.3 vs. 93.8, 21.7, and 8.1), respectively, using the pseudovirus neutralizing antibody reaction. The ID50s of BA.5, BQ.1.1, and XBB.1 in group 2 were 4.9, 35.0, and 72.5 times lower than that of D614G (1978.3 vs. 400.7, 56.5, and 27.3), respectively. The ID50s of BA.5, BQ.1.1, and XBB.1 in group 3 were 4.1, 21.3, and 65.9 times lower than that of D614G (2681.8 vs. 657.4, 126.2, and 40.7), respectively (92). The mRNA vaccine, when combined with the NVX-CoV2373 vaccine, moderately increased the humoral immunity against BA.5, BQ.1.1, and XBB.1. In all three groups, the neutralizing antibody titer against XBB.1 was low but slightly increased in group 3.

Wang et al. studied serum samples collected within 14 days after vaccination with two doses of CoronaVac and one dose of ZF2001 vaccine. The ID50s of BA.5, BF.7, BQ.1, BQ.1.1, XBB.1, and XBB.1.5 were 16, 16, 56, 56, 52, and 56 times lower than that of the WT (D614G) (724 vs. 44, 44, 13, 13, 14, and 13), respectively. Serum samples were collected within 14 days after inoculation with three doses of CoronaVac. The ID50s of BA.5, BF.7, BQ.1, BQ.1.1, XBB.1, and XBB.1.5 were 16, 15, 29, 27, 29, and 40 times lower than that of the WT (D614G) (516 vs. 32, 35, 18, 19, 18,and 13) (84). Compared with three doses of CoronaVac, two doses of CoronaVac and one1 dose of ZF2001 vaccine had no significant effect on inducing higher neutralizing antibody titers against the new mutant strains.

Zuo et al. (83) conducted a study to evaluate one dose of mRNA vaccine after two doses of inactivated vaccine (group 1) and one to two doses of mRNA vaccine after three doses of inactivated vaccine (group 2). The researchers employed ELISA and lentivirus-based pseudovirus neutralization tests to assess the immune response. Serum samples were collected within one to five months after injection. In group 1, the NT50 values of BA.5, BF.7, BQ.1.1, and XBB.1 were 4.54, 6.72, 30.98, and 99.42 times lower than those of G614 (2386 vs. 526, 355, 77, and 24). The NT50 values of BA.5, BF.7, BQ.1.1, and XBB.1 in group 2 were 2.61, 3.48, 16.63, and 110.6 times lower than those in G614 (2212 vs. 848, 635, 133, and 20). In this experiment, three doses of mRNA vaccine (group 3) were also evaluated, and the NT50 values of BA.5, BF.7, BQ.1.1, and XBB.1 were 3.11, 5.91, 17.68, and 50.36 times lower than G614 (1662 vs. 535, 281, 94, and 33), respectively. Three doses of inactivated vaccine (Group 4) were also delivered. The NT50 values of BA.5, BF.7, BQ.1.1, and XBB.1 were 2.04, 3.13, 5.88, and 7.83 times lower than G614 (47 vs. 23, 15, 8, and 6), respectively. In group 1, 2, and 3, the levels antibodies against all mutants were higher than those in group 4 (inoculated with three doses of inactivated vaccine). For BA.5, BF.7, and BQ.1.1, one to two doses of mRNA vaccine after three doses of inactivated vaccine could induce more antibodies, while for the updated mutant XBB.1, three doses of mRNA vaccine produced more neutralizing antibodies than the other two groups; however, in the four groups of data, the number of neutralizing antibodies against XBB.1 was very low.

## Vaccine effectiveness in preclinical trials

The Omicron sub-variant emerged as the epidemic progressed, and ongoing efforts are being made to develop new vaccines. In the field of protein vaccines, some experiments have been based on animal studies. Specific experiments have yielded positive results in animals and have potential value for future epidemics. The vaccine efficacy (VE) in the different mutant preclinical trials is shown in **Table S3**.

Li et al. designed a recombinant protein vaccine consisting of a homotype RBD dimer immunogen and a heterotype chimeric RBD dimer. This experiment compared the neutralizing activity of five RBD dimer vaccines in terms of mouse serum antibody titers against BA.5, BF.7, XBB, BQ.1, and BQ.1.1 mutants. In the PT BD Homodimer (PT-PT) group, the neutralizing antibody titers of 50% pseudovirus against the BA.5, BF.7, XBB, BQ.1, and BQ.1.1 mutants were 50.48, 844.21, 9417.64, 503.92, and 6726.89 times (423794 vs. 8395, 423794 vs. 502, 423794 vs. 45, 423794 vs. 841, and 423794 vs. 63) lower than those of the PT, respectively. In the BA.2 RBD Homodimer (BA.2-BA.2) group, the neutralizing antibody titers of 50% pseudovirus against the BA.5 and BF.7 mutants were 10.21 and 3.47 times (158775 vs. 15545,53968 vs. 15545) higher than those of the PT, respectively. The neutralizing antibody titers of the XBB, BQ.1 and BQ.1.1 mutants were 39.55, 11.79, and 11.37 times (15545 vs. 393, 1318, and 1367) lower than that of the PT, respectively. In the PT-Beta RBD Heterodimer (PT-Beta) group, the neutralizing antibody titers of 50% pseudovirus against the BA.5, BF.7, XBB, BQ.1, and BQ.1.1 mutants were 12.45, 62.57, 3654, 759.04, and 2182.25 times (157122 vs. 12621, 2511, 43, 207, and 72) lower than those of the PT, respectively. In the data of the Delta-BA.1 RBD Homodimer (DeltaBA.1) group, the neutralizing antibody titers of 50% pseudovirus against the BA.5, BF.7, XBB, BQ.1, and BQ.1.1 mutants were 6.55, 212.44, 815.64, 56.80, 382.72 times (248771 vs. 37962, 1171, 305, 4380, and 650) lower than those of the PT, respectively. Finally, in the data of the Delta-BA.2 RBD Heterodimer (Delta-BA.2) group, the neutralizing antibody titers of 50% pseudovirus against the BA.5, BF.7, XBB, BQ.1, and BQ.1.1 mutants were reduced by 3.51, 14.30, 697.78, 148.25, and 416.73 times (210033 vs. 58771, 14688, 301, 1412, and 504), respectively. In this animal experiment, the Delta–Omicron BA.1 group and the BA.2 RBD heterodimer group showed strong neutralizing activity against the emerging Omicron variants (61).

In addition, in a ferritin-based COVID-19 nanoparticle vaccine, Weidenbacher et al. invented a protein nanoparticle vaccine called DCFHP. In the Rhesus macaque experiment, injection was performed on day 0, and a second injection was performed on day 92. The NT50 data of the BA.5, BQ.1, and BQ.1.1 mutants in this group were 31.63, 63.11, and 50.14 times lower than those of the PT (25118 vs. 794, 398, and 501), respectively (100). The protein nanoparticle vaccine exhibited characteristics that make it easily absorbed by antigen–presenting dendritic cells (101), and its unique antigen multivalent presentation facilitates receptor clustering and subsequent B-cell activation (102). Furthermore, the ferritin–based V-2 vaccine has demonstrated safety and efficacy in clinical trials (103). Besides its strong neutralizing activity against BA.5, the DCFHP vaccine also offers advantages such as low cost, large-scale production, and broad-spectrum coverage. Consequently, it might be suitable for use in pediatric populations, including infants.

## Conclusions and prospects

Although the end of COVID-19 remains uncertain, its continuous development poses a significant global health threat. However, the development of vaccines has instilled hope worldwide. Currently, vaccination stands as the most effective measure against COVID-19. It is worth noting that after vaccination, the antibody levels in the body against different variants of the novel coronavirus will continue to decline so that people will be at risk of reinfection. The research and development of vaccines should focus on reducing the severity and mortality of COVID-19 infection while ensuring safety and playing a preventive role. Previous experiences with vaccine development against coronavirus strains such as SARS-CoV and MERS-CoV have meant that international cooperation with specific goals, substantial financial investment, and the dedicated efforts of many scientists have led to the emergence of hundreds of different vaccines within just one year (104).

In this review, we assessed the efficacy of recently approved vaccines against neutralizing antibodies targeting various lineages of the latest Omicron variant. We also reviewed the effectiveness of heterologous vaccination and preclinical multivalent vaccines. However, the ongoing global COVID-19 pandemic means that there is an urgent requirement for a universal vaccine against the novel coronavirus that demonstrates both high immunogenicity and safety. The development of universal vaccines is related to the diversity and continuous changes of SARS-CoV-2 mutant strains and the final efficacy of vaccines, which might affect the duration of protection, quality monitoring, market supervision, and other issues (105).

## Author contributions

KC: Funding acquisition, Writing – original draft, Writing – review & editing. LZ: Data curation, Formal analysis, Resources, Software, Visualization, Writing – original draft, Writing – review & editing. ZF: Conceptualization, Data curation, Formal analysis, Investigation, Software, Writing – original draft, Writing – review & editing. JXL: Data curation, Formal analysis, Writing – original draft. CL: Data curation, Formal analysis, Writing – original draft. WS: Data curation, Writing – original draft. ZH: Software, Writing – original draft. RC: Software, Writing – original draft. YZ: Funding acquisition, Writing – original draft, Writing – review & editing. JHL: Funding acquisition, Writing – original draft, Writing – review & editing.
