# Supplementary material for: Analysis of the protective efficacy of approved COVID-19 vaccines against Omicron variants and the prospects for universal vaccines
Source: Front Immunol. 2023 Nov 27;14:1294288. doi: 10.3389/fimmu.2023.1294288 (PMC10711607; doi:10.3389/fimmu.2023.1294288)
Supplement: Supplementary file 1 [file Table_1.docx]

**TABLE S1 | Approved vaccine information**

| **Vaccine type** | **Research and development enterprises or institutions** | **Dose/interval** | **Clinical batch number** | **Phase** |
| --- | --- | --- | --- | --- |
| Protein Subunit | Novavax: Nuvaxovid | 2/21 | NCT05372588 | 3 |
|  | Razi Vaccine and Serum Research Institute: Razi Cov Pars | 3/21/51 | IRCT20210206050259N3 | 3 |
|  | Serum Institute of India: COVOVAX (Novavax formulation) | 2/28 | NCT05433285 | 3 |
|  | Sanofi/GSK: VidPrevtyn Beta | 2/21 | NCT05124171 | 3 |
|  | Livzon Mabpharm Inc: V-01 | 2/21 | NCT05096832 | 3 |
|  | Medigen: MVC-COV1901 | 2/28 | NCT05011526 | 3 |
|  | PT Bio Farma: IndoVac | 2/28 | NCT05433285 | 3 |
|  | Anhui Zhifei Longcom: Zifivax |  | NCT05616754 | 3 |
|  | Bagheiat-allah University of Medical Sciences: Noora vaccine | 3/21/35 | IRCT20210620051639N3 | 3 |
|  | Vector State Research Center of Virology and Biotechnology: EpiVacCorona | 2/21 | NCT04780035 | 3 |
|  | Biological E Limited: Corbevax | 2/28 | CTRI/2021/08/036074 | 3 |
|  | Vaxine/CinnaGen Co.: SpikoGen | 2/21 | IRCT20150303021315N24 | 3 |
|  | Takeda: TAK-019 (Novavax formulation) |  | NCT05299359 | 3 |
|  | Center for Genetic Engineering and Biotechnology (CIGB): Abdala | 3/14/28/or | RPCEC00000359 | 3 |
|  | SK Bioscience Co Ltd: SKYCovione | 2/28 | NCT05007951 | 3 |
|  | National Vaccine and Serum Institute: Recombinant SARS-CoV-2 Vaccine (CHO Cell) |  | NCT05069129 | 3 |
| Virus-like particles (VLP) | Medicago: Covifenz | 2/21 | NCT05040789 | 3 |
|  | Moderna: Spikevax Bivalent Original/Omicron BA.1 |  | NCT05543356 | 3 |
| DNA-based | Zydus Cadila: ZyCoV-D | 3/28/56 | CTRI/2022/06/043365 | 3 |
| RNA-based | Walvax: AWcorna | 2/14/28 | NCT04847102 | 3 |
|  | Moderna: Spikevax Bivalent Original/Omicron BA.1 | 2/21 | NCT05543356 | 3 |
|  | Moderna: Spikevax Bivalent Original/Omicron BA.4/BA.5 | 1/0 | NCT04927065 | 2/3 |
|  | Gennova Biopharmaceuticals Limited: GEMCOVAC-19 | 2/28 | CTRI/2022/04/041880 | 2/3 |
|  | Pfizer/BioNTech: Comirnaty Bivalent Original/Omicron BA.4/BA.5 | 2/21 | NCT05543616 | 2/3 |
|  | Pfizer/BioNTech: Comirnaty | 2/21 | NCT05543356 | 3 |
|  | Moderna: Spikevax | 2/21 | NCT05543356 | 3 |
|  | Pfizer/BioNTech: Comirnaty Bivalent Original/Omicron BA.1 | 2/21 | NCT05543356 | 3 |
| Non-Replicating Viral Vector | Gamaleya: Sputnik Light | 2/21 | CTRI/2022/04/041792 | 3 |
|  | Bharat Biotech: iNCOVACC | 1/0 | CTRI/2022/02/040065 | 3 |
|  | CanSino: Convidecia | 2/21 | NCT05442684 | 3 |
|  | CanSino: Convidecia Air | 2/21 | NCT05442684 | 3 |
|  | Gamaleya: Sputnik V | 2/21 | NCT04530396 | 3 |
|  | Janssen (Johnson & Johnson): Jcovden | 1-2/56 | NCT05048940 | 3 |
|  | Serum Institute of India: Covishield (Oxford/ AstraZeneca formulation) | 2/21 | CTRI/2022/04/042017 | 3 |
| Inactivated | Valneva: VLA2001 | 2/21 | NCT04956224 | 3 |
|  | Shifa Pharmed Industrial Co: COVIran Barekat | 2/14 | IRCT20201202049567N3 | 2/3 |
|  | Shifa Pharmed Industrial Co: COVIran Barekat | 2/14 | IRCT20201202049567N3 | 2/3 |
|  | Bharat Biotech: Covaxin | 2/14 | CTRI/2022/02/040065 | 3 |
|  | Health Institutes of Turkey: Turkovac | 2/21 | NCT04942405 | 3 |
|  | Research Institute for Biological Safety Problems (RIBSP): QazVac | 2/21 | NCT04691908 | 3 |
|  | Shenzhen Kangtai Biological Products Co: KCONVAC | 2/28 | NCT04852705 | 3 |
|  | Sinovac: CoronaVac | 2/14 | NCT05433272 | 3 |
|  | Sinopharm (Beijing): Covilo | 2/28 | NCT05374954 | 3 |
|  | Sinopharm (Wuhan): Inactivated (Vero Cells) | 2/21 | ChiCTR2000034780 | 3 |

| **TABLE S2 \| The vaccine effectiveness (VE) ofheterologous vaccination on different mutants.** | | | | | | | |
| --- | --- | --- | --- | --- | --- | --- | --- |
|  | **BA.5** | **BF.7** | **XBB** | **XBB.1** | **XBB.1.5** | **BQ.1** | **BQ.1.1** |
| **heterologous vaccination** |  |  |  |  |  |  |  |
| Vaccines 3 doses  (CoronaVac *2+BNT162 b2 ) | -11.09times  ( pVNT50，WT，pVNT，1475 vs. 133)(1) | -21.07times  (pVNT50，WT，pVNT，1475 vs. 70)(1) | -47.58times  (pVNT50，WT，pVNT，1475 vs. 31)(1) |  | -46.09times  (pVNT50，WT，pVNT，1475 vs. 32)(1) | -28.36times  (pVNT50，WT，pVNT，1475 vs. 52)(1) | -34.30 times  (pVNT50，WT，pVNT，1475 vs. 43)(1) |
| Vaccines 2doses  (CoronaVac *2+Ad5-nCov2 *1 ) | -8.4times  ( pVNT50，WT，pVNT，3150 vs. 375)(1) | -18.10tims  (pVNT50，WT，pVNT，3150 vs. 174)(1,1) | -71.59times  (pVNT50，WT，pVNT，3150 vs. 44)(1) |  | -73.25times  (pVNT50，WT，pVNT，3150 vs. 43)(1) | -19.56times  (pVNT50，WT，pVNT，3150 vs. 161)(1) | -26.03 times  (pVNT50，WT，pVNT，3150 vs. 121)(1) |
| Vaccines 4 doses  (CoronaVac *3+Ad5-nCov2 *1 ) | -4.14times  ( pVNT50，WT，pVNT，943 vs. 228)(1) | -6.93 times  (pVNT50，WT，pVNT，943 vs. 136)(1) | -22.45times  (pVNT50，WT，pVNT，943 vs. 42)(1) |  | -26.19times  (pVNT50，WT，pVNT，943 vs. 36)(1) | -9.82times  (pVNT50，WT，pVNT，943 vs. 96)(1) | -12.74 times  (pVNT50，WT，pVNT，943 vs. 74)(1) |
| Vaccines 2 doses  (Ad5-nCoV *1  +mRNA-1273 *1) | -3.10times  (IC50，B.1.189，MNA，221.30 vs. 71.36)(2) |  |  | -34.09 times  (IC50，B.1.189，MNA，221.30 vs. 6.49)(2) |  |  |  |
| Vaccines 3 doses  (mRNA vaccine*2+SCTv01C 20μg *1，for men) | +1.46times  (pVNT50，BA.1，pVNT，1736 vs. 1189)(3) |  |  |  |  |  |  |
| Vaccines 3 doses  (mRNAvaccine  *2+SCTv01E 30μg *1，for men) | +1.37times  (pVNT50，BA.1，pVNT，2281 vs. 1659)(3) |  |  |  |  |  |  |
| Vaccines 2or 3 doses  (Ad26.COV2.S  *1or 2+NVX-CoV2373 *1) | -5.3times  (ID50，D614G，PsVNA，499.3 vs. 93.8)(4) |  |  | -61.6times  (D50，D614G，PsVNA，499.3 vs. 8.1)(4) |  |  | -23times  (D50，D614G，PsVNA，499.3 vs. 21.7)(4) |
| Vaccines 3 doses  (mRNA-1273 100mcg *2+NVX-CoV2373 *1) | -4.9times  (ID50，D614G，PsVNA，1978.3 vs. 400.7)(4) |  |  | -72.5times  (ID50，D614G，PsVNA，1978.3 vs. 27.3)(4) |  |  | -35times  (D50，D614G，PsVNA，1978.3 vs. 56.3)(4) |
| Vaccines 3 doses  (BNT162b 30-mcg *2+NVX-CoV2373 *1) | -4.1times  (ID50，D614G，PsVNA，2681.8 vs. 657.8)(4) |  |  | -65.9times  (ID50，D614G，PsVNA，2681.8 vs. 40.7)(4) |  |  | -21.3times  (D50，D614G，PsVNA，2681.8 vs. 126.2)(4) |
| Vaccines 3 doses  (CoronaVac *2+ZF2001*1) | -16times  (ID50，D614G，pVNT，14d，724 vs. 44)(5) | -16times  (ID50，D614G，pVNT，14d，724 vs. 44)(5) |  | -52times  (ID50，D614G，pVNT，14d，724 vs. 14)(5) | -56 times  (ID50，D614G，pVNT，14d，724 vs. 13)(5) | -56times  (ID50，D614G，pVNT，14d，724 vs. 13)(5) | -56times  (ID50，D614G，pVNT，14d)，724 vs. 13)(5) |
| Vaccines 3 doses  (Inactivated Vaccines*2+mRNA vaccine booster *1) | -4.54times  (pVNT50，G614，pVNT，1-5month，2386 vs. 526)(6) | -6.72times  (pVNT50，G614，pVNT，1-5month，2386 vs. 355)(6) |  | -99.42times  (pVNT50，G614，pVNT，1-5month，2386 vs. 24)(6) |  |  | -30.98imes  (pVNT50，G614，pVNT，1-5month，2386 vs. 77)(6) |
| Vaccines 4or5 doses  (Inactivated Vaccines  *3+mRNA vaccine booster *1 or 2 ) | -2.61times  (pVNT50，G614，pVNT，1-5month，2212 vs. 848)(6) | -3.48times  (pVNT50，G614，pVNT，1-5month2212 vs. 635)(6) |  | -110.6times  (pVNT50，G614，pVNT，1-5month，2212 vs. 20)(6) |  |  | -16.63times  (pVNT50，G614，pVNT，1-5month，2212 vs. 133)(6) |

*1 dose: The vaccine was immunized once. 2 doses were immunized twice. 3 doses were immunized twice third. 4 doses. were immunized twice fourth. Booster dose, Vaccines to strengthen the needle.IC50, 50% true virus neutralization titer; ID50, Infection dose 50. pVNT50, 50% pseudovirus neutralization tite. PRNT50, 50% plaque reduction neutralization test. D614G, Taking the D614 G mutant as the reference object. WT, Take the wild-type (Wuhan-hu-1)、SARS-CoV-2 prototype（PT）、 UT-NC002-1T、B.1.1 as the reference. The new variant strain‘s GMT compared with the comparative strain'sGMT, with a ratio greater than 0 and a positive multiple; The comparative strain‘s GMT compared with the new variant strain‘s GMT, with a ratio less than 0 and a negative multiple. Greater than 0 times (green ), 0-10 times (yellow ), -10-50 times (blue ), -50-100 times (purple ), greater than -100 times (red ), vaccine effectiveness（orange ).1-5month:1-5month after administration,33-57days:33-57days, 23-94days after administration:23-94days after administration.

| **TABLE S3 \| The vaccine effectiveness (VE) of pre-clinical trials on different mutants.** | | | | | | |
| --- | --- | --- | --- | --- | --- | --- |
|  | **BA.5** | **BF.7** | **XBB** | **XBB.1** | **BQ.1** | **BQ.1.1** |
| **Vaccine efficacy in preclinical trials** |  |  |  |  |  |  |
| Vaccine 1dose  ( PT RBD Homodimer vaccine) | -50.48times  ( pVNT50，PT，pVNT，in Mice，423794 vs. 8395 )(7) | -844.21times  ( pVNT50，PT，pVNT，in Mice，423794 vs. 502)(7) | -9417.64times  ( pVNT50，PT，pVNT，in Mice，423794 vs. 45)(7) |  | -503.92times  ( pVNT50，PT，pVNT，in Mice，423794 vs. 841)(7) | -6726.89times  ( pVNT50，PT，pVNT，in Mice，423794 vs. 63)(7) |
| Vaccine 1dose  ( BA.2 RBD Homodimer vaccine) | +10.21times  ( pVNT50，PT，pVNT，in mice，158775 vs. 15545  )(7) | +3.47times( pVNT50，PT，pVNT，in mice，53968 vs.  15545)(7) | -39.55times( pVNT50，PT，pVNT，in Mice，15545 vs. 393)(7) |  | -11.79mes  ( pVNT50，PT，pVNT，in Mice，15545 vs. 1318)(7) | -11.37times(  pVNT50，PT，pVNT，in Mice，15545 vs. 1367)(7) |
| Vaccine 1dose  ( PT-Beta RBD Homodimer vaccine) | -12.45times  ( pVNT50，PT，pVNT，in Mice，157122 vs. 12621)(7) | -62.57times  ( pVNT50，PT，pVNT，in Mice，157122 vs. 2511)(7) | -3654times  ( pVNT50，PT，pVNT，in Mice，157122 vs. 43)(7) |  | -759.04times  ( pVNT50，PT，pVNT，in Mice，157122 vs. 207)(7) | -2182.25times  ( pVNT50，PT，pVNT，in Mice，157122 vs. 72)(7) |
| Vaccine 1dose  ( Delta-BA.1 RBD Homodimer vaccine) | -6.55times  ( pVNT50，PT，pVNT，in Mice,，248771 vs. 37962)(7) | -212.44times  ( pVNT50，PT，pVNT，in Mice，248771 vs. 1171)(7) | -815.64times  ( pVNT50，PT，pVNT，in Mice，248771 vs. 305)(7) |  | -56.80times  ( pVNT50，PT，pVNT，in Mice，248771 vs. 4380)(7) | -382.72times  ( pVNT50，PT，pVNT，in Mice，248771 vs. 650)(7) |
| Vaccine 1dose  (Delta~BA.2 RBD Heterodimer ) | -3.51times( pVNT50，PT，pVNT，in Mice，210033 vs. 59771)(7) | -14.30times( pVNT50，PT，pVNT，in Mice，210033 vs. 14688)(7) | -697.78times( pVNT50，PT，pVNT，in Mice，210033 vs. 301)(7) |  | -148.25times( pVNT50，PT，pVNT，in Mice，210033 vs. 1412)(7) | -416.73times( pVNT50，PT，pVNT，in Mice，210033 vs. 504)(7) |
| Vaccine 3doses  ( RBDM 25μg vaccine*3) | -3.64times  ( IC50，WT，FRNT，in Rhesus macaques，1899 vs. 521)(8) |  |  | -18.61times  ( IC50，WT，FRNT，in Rhesus macaqu  -es，1899 vs. 102)(8) |  |  |
| Vaccine 3doses  ( RBDM 50μg vaccine*3) | -3.16times  ( IC50，WT，FRNT，in Rhesus macaques，1632 vs. 517)(8) |  |  | -15.84times  ( IC50，WT，FRNT，in Rhesus macaqu  -es，1632 vs. 103)(8) |  |  |
| Vaccine 2doses  ( DCFHP-alum vaccine*2) | -31.63times  ( NT50，WT，FRNT，inRhesus macaques，92D，25118 vs. 794)(9) |  |  |  | -63.11times  ( NT50，WT，FRNT，in Rhesus macaques，92D，25118 vs. 398)(9) | -50.14times  ( NT50，WT，FRNT，in Rhesus macaques，92D，25118 vs. 501)(9) |

*1 dose: The vaccine was immunized once. 2 doses were immunized twice. 3 doses were immunized twice third. 4 doses. were immunized twice fourth. Booster dose, Vaccines to strengthen the needle.IC50, 50% true virus neutralization titer; ID50, Infection dose 50. pVNT50, 50% pseudovirus neutralization tite. PRNT50, 50% plaque reduction neutralization test. D614G, Taking the D614 G mutant as the reference object. WT, Take the wild-type (Wuhan-hu-1)、SARS-CoV-2 prototype（PT）、 UT-NC002-1T、B.1.1 as the reference. The new variant strain‘s GMT compared with the comparative strain'sGMT, with a ratio greater than 0 and a positive multiple; The comparative strain‘s GMT compared with the new variant strain‘s GMT, with a ratio less than 0 and a negative multiple. Greater than 0 times (green ), 0-10 times (yellow ), -10-50 times (blue ), -50-100 times (purple ), greater than -100 times (red ), vaccine effectiveness（orange ).1-5month:1-5month after administration,33-57days:33-57days, 23-94days after administration:23-94days after administration.

**References**

1. Zhu A, Wei P, Man M, Liu X, Ji T, Chen J, Chen C, Huo J, Wang Y, Zhao J. Antigenic characterization of SARS-CoV-2 Omicron subvariants XBB.1.5, BQ.1, BQ.1.1, BF.7 and BA.2.75.2. *Signal Transduct Target Ther* (2023) 8:125. doi: 10.1038/s41392-023-01391-x

2. Neutralization of Omicron BA.1, BA.5.1.6, BQ.1.3 and XBB1.1 induced by heterologous vaccination Ad5-nCoV and mRNA-1273 - PubMed. https://pubmed.ncbi.nlm.nih.gov/37120638/ [Accessed August 30, 2023]

3. Safety and immunogenicity of a tetravalent and bivalent SARS-CoV-2 protein booster vaccine in men - PubMed. https://pubmed.ncbi.nlm.nih.gov/37422518/ [Accessed August 30, 2023]

4. Immunogenicity of NVX-CoV2373 heterologous boost against SARS-CoV-2 variants - PubMed. https://pubmed.ncbi.nlm.nih.gov/37433788/ [Accessed August 30, 2023]

5. Neutralization of SARS-CoV-2 BQ.1.1, CH.1.1, and XBB.1.5 by breakthrough infection sera from previous and recent waves in China - PubMed. https://pubmed.ncbi.nlm.nih.gov/37369648/ [Accessed August 30, 2023]

6. Heterologous inactivated virus/mRNA vaccination response to BF.7, BQ.1.1, and XBB.1 - PubMed. https://pubmed.ncbi.nlm.nih.gov/37069895/ [Accessed August 30, 2023]

7. Li D, Duan M, Wang X, Gao P, Zhao X, Xu K, Gao GF. Neutralization of BQ.1, BQ.1.1, and XBB with RBD-Dimer Vaccines. *N Engl J Med* (2023) 388:1142–1145. doi: 10.1056/NEJMc2216233

8. An RBD virus-like particle vaccine for SARS-CoV-2 induces cross-variant antibody responses in mice and macaques - PubMed. https://pubmed.ncbi.nlm.nih.gov/37120453/ [Accessed August 30, 2023]

9. A ferritin-based COVID-19 nanoparticle vaccine that elicits robust, durable, broad-spectrum neutralizing antisera in non-human primates - PubMed. https://pubmed.ncbi.nlm.nih.gov/37069151/ [Accessed August 30, 2023]
